# CogniPaz, an Ecologically Oriented Gamified mHealth Tool for Detecting Mild Cognitive Impairment: Prospective Cohort Diagnostic Accuracy Study

**DOI:** 10.2196/99688

**Published:** 2026-11-04

**Authors:** Carlos Estebas-Armas, Alejandro Medina-López, Daniel Blanco-de-Miguel, Itsaso Losantos-García, María Hernández-Barral, Elisa Alonso-López, Sergio Henche-Rodríguez, Oscar Ocampo-Pérez, Gerardo Ricardo Zmork-Martínez, María Alonso-de-Leciñana, Ana Frank-García, Ángel Martín-Montes

**Affiliations:** 1 Department of Neurology and Stroke Center, La Paz University Hospital, Universidad Autónoma de Madrid, Hospital La Paz Institute for Health Research, Paseo de la Castellana, 241, Madrid, 28046, Spain, 34 677396884; 2 Biostatistics Department, La Paz University Hospital, Universidad Autónoma de Madrid, Hospital La Paz Institute for Health Research, Madrid, Spain; 3 Networking Research Center on Neurodegenerative Diseases (CIBERNED), Instituto de Salud Carlos III, Madrid, Spain

**Keywords:** mild cognitive impairment, amnestic mild cognitive impairment, subjective cognitive decline, digital biomarkers, cognitive assessment, mobile applications, early diagnosis, Alzheimer disease, neurodegenerative diseases, ecological validity, usability

## Abstract

**Background:**

Discriminating mild cognitive impairment (MCI) from subjective cognitive decline (SCD), and healthy controls (HC) remains challenging. Digital tools may overcome paper-and-pencil screening’s limitations.

**Objective:**

This study evaluated the diagnostic accuracy and usability of CogniPaz (Daniel Blanco), an ecologically oriented, mobile health (mHealth), gamified cognitive assessment tool exploring memory and executive functions, in discriminating MCI (particularly amnestic phenotypes [aMCI]) from SCD, and HC.

**Methods:**

This prospective observational study recruited a convenience sample of consecutive patients with cognitive complaints and HC at La Paz University Hospital Cognitive Disorders Unit from April 2025 to January 2026. Demographic and clinical characteristics, and Montreal Cognitive Assessment (MoCA) scores were collected. An evaluator blinded to the MoCA scores oversaw the automated, self-administered CogniPaz assessment. Participants were classified into HC (no cognitive complaints, MoCA score≥26), SCD (cognitive complaints, MoCA score≥26), and MCI (cognitive complaints without functional impairment, MoCA score<26, subdivided into amnestic [aMCI] and nonamnestic phenotypes). Receiver operating characteristic (ROC) analyses using age-adjusted models, the DeLong test, and bootstrap validation evaluated diagnostic accuracy across groups. The correlation between MoCA and CogniPaz scores was assessed using Spearman rank correlation. Participants with available biomarker data underwent exploratory analysis of CogniPaz’s ability to detect biologically confirmed any-cause neurodegeneration (bND). Usability was tested using an ad hoc Likert questionnaire (1‐9 points).

**Results:**

The participants (n=84, 57.1% female; median age 72.0, IQR 62.5-77.0 years) included 27 HC, 17 with SCD, 22 with aMCI, and 18 with nonamnestic MCI. The aMCI group was older than the SCD group (age: median 76.5, IQR 70.8-78.8 vs median 59.0, IQR 50.0-69.0 years; Kruskal-Wallis/Dunn, *P*=.003). CogniPaz scores correlated with MoCA scores (ρ*=*0.81, 95% CI 0.75‐0.89; *P<*.001). CogniPaz showed good concurrent accuracy, independently of age: MCI vs HC (area under the curve [AUC]=0.880, 95% CI 0.798‐0.961), MCI vs SCD (AUC=0.915, 95% CI 0.841‐0.990), aMCI vs HC (AUC=0.910, 95% CI 0.830‐0.990), and aMCI vs SCD (AUC=0.943, 95% CI 0.878‐1.000). At the provisional <28.5 cutoff point, sensitivity ranged from 85.0% (34/40, 95% CI 70.2‐94.3) to 90.9% (20/22, 95% CI 70.8‐98.9) and specificity from 74.1% (20/27, 95% CI 53.7‐88.9) to 82.4% (14/17, 95% CI 56.6‐96.2) across comparisons. CogniPaz suggested capacity to detect bND (AUC=0.810, 95% CI 0.661‐0.958), with potential incremental value over MoCA and age (odds ratio [OR]=0.78, 95% CI 0.60‐0.94; *P*=.02). Median administration time was 9.39 (IQR 7.53‐11.38) minutes with 100% (84/84) completion. User satisfaction was high (median score 8.4/9, IQR 7.9‐8.6; Cronbach α=0.84).

**Conclusions:**

CogniPaz is an innovative, user-friendly mHealth tool that addresses classical cognitive screening tools’ limitations through a brief, multidomain, minimally supervised gamified format, with high patient acceptance. It correlates with MoCA scores, shows good concurrent capacity to differentiate MCI, particularly aMCI, from SCD and HC, and appears promising for identifying bND. Pending external longitudinal validation, these findings support CogniPaz as a candidate for early cognitive screening in primary care.

## Introduction

Mild cognitive impairment (MCI) is a transitional clinical state between normal aging and dementia, characterized by objective cognitive decline with largely preserved functional independence [1]. Within MCI, amnestic forms (aMCI) are associated with an increased risk of progression to dementia in Alzheimer disease (AD) [2]. Subjective cognitive decline (SCD) refers to a self-perceived, persistent decline in cognitive capacity, typically involving memory, despite normal performance on standard cognitive tests, and is now considered a risk state for future MCI and dementia [3]. Differentiating aMCI from SCD is clinically relevant but challenging because complaints and even subtle performance differences frequently overlap, and early deficits may be masked by cognitive reserve. Early and accurate identification of individuals at increased risk for neurodegenerative disease is essential to guide monitoring, risk factor management, and potential disease-modifying interventions.

Traditional paper-and-pencil screening tools, such as the Mini-Mental State Examination (MMSE) and the Montreal Cognitive Assessment (MoCA), have important roles but can exhibit ceiling effects, limited sensitivity to early changes, and constraints in repeated administration [4,5]. These limitations have prompted a rapid shift toward digital tools for cognitive assessments. Recent narrative and systematic reviews indicate substantial growth in digital cognitive tools for detecting MCI and dementia, including web-based batteries, tablet-based neuropsychological platforms, and smartphone apps [6-9]. Digital assessments can improve standardization, capture reaction times, automate scoring, and facilitate remote or high-frequency testing. Moreover, the scalability of these tools makes them ideal candidates to meet the growing demand for screening in aging populations [5]. Indeed, the integration of emerging digital technologies is increasingly recognized as a crucial strategy for developing scalable algorithms that facilitate early AD diagnosis and provide globally accessible screening guidance [10-12]. Meta-analytic evidence suggests that digital cognitive tools generally achieve good sensitivity and specificity for MCI and dementia detection, but performance varies by domain, platform, and comparison group [13-16].

However, important limitations remain. Recent reviews show that although digital cognitive assessment for older adults has expanded quickly, validation remains uneven: across web-, tablet-, and smartphone-based tools, construct validity and robust normative data are still incompletely documented [5,17]. Usability and implementation barriers remain a recurring problem, including difficulties navigating software, unstable connectivity, lengthy assessment times, and limited adaptability for people with less education and lower digital literacy [18,19].

These gaps support the development of brief, user-friendly tools that can be deployed in real-world pathways. Many digital tools have been validated primarily to distinguish dementia or global cognitive impairment from normal cognition, rather than to separate SCD from MCI in real-world memory clinic populations [5,20]. Ecological validity and user engagement, particularly among older patients and patients with anxiety, are often underreported, and the integration of digital tests into existing pathways for specialized care is still evolving [5,14,19,20]. Moreover, there is a paucity of tools capable of exploring executive functions alongside episodic memory in a brief, gamified format that can be administered easily by nonspecialist personnel [21]. In this regard, incorporating principles from adaptive mobile health (mHealth) systems, which use human-centered design to dynamically adjust to the user’s needs and have proven effective in improving engagement and usability [22,23], offers a valuable framework for developing cognitive tools tailored to older adults.

To address these needs, we developed CogniPaz (Daniel Blanco), an ecologically oriented, tablet-based, gamified digital cognitive screening tool designed for rapid administration by nonspecialist clinicians (eg, general physicians or nurses) as a screening tool to identify MCI in patients consulting for SCD. Accordingly, the primary objectives of this prospective study were to evaluate the diagnostic accuracy of CogniPaz in discriminating MCI, particularly aMCI, from SCD and healthy controls (HC), to examine its correlation with MoCA scores, and to assess its feasibility and usability. In a secondary exploratory analysis, we also evaluated whether CogniPaz could identify biologically confirmed any-cause neurodegeneration (bND) in a subset of participants with available biomarker data.

## Methods

### Instrumentation

CogniPaz was designed as a digital cognitive battery test. The study version of CogniPaz was implemented as an Android tablet app using Ionic/Angular and Capacitor. The protocol comprised 9 tasks, yielding a maximum total score of 38 points (Figure 1).

The device automatically provided the initial instructions to the participants before they began the tasks. To ensure accessibility, the platform featured an intuitive visual interface with minimal text to reduce linguistic bias, large font sizes for mild visual impairment, and an integrated audio guide for clear instructions. The assessment is structured as follows:

**Figure 1.**
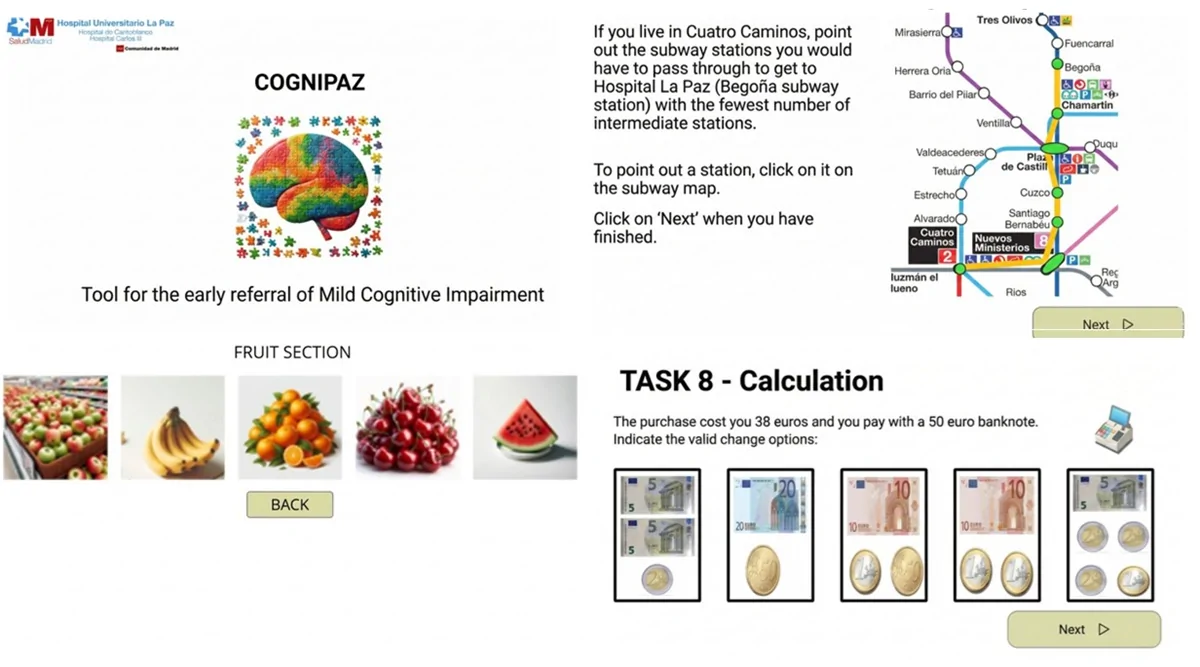
Examples of tasks included in the CogniPaz cognitive screening tool (English translation). The complete battery, in its original Spanish version, is provided in Multimedia Appendix 1.

### Attention and Orientation (16 Points)

#### Task 1: Verbal Attention With Visual Input-Encoding

The participants encoded a list of 6 words (salmón [salmon], hamburguesas [hamburgers], azúcar [sugar], leche [milk], plátanos [bananas], and lejía [bleach]) presented sequentially in full-screen mode at 2-second intervals. This task was performed across 2 trials; after each presentation, participants verbally recalled the items using the tablet microphone; speech was processed transiently, and the resulting text was scored automatically by the app (6+6 points).

#### Task 3.1: Verbal Attention With Auditory Input-Encoding

The participants listened to a simulated TV news broadcast regarding a fire. Immediately after, they were required to manually type 3 specific details regarding the news story, including the number of victims, the distance in meters from the fire station, and the time of the event. They were also explicitly instructed to memorize these details, as they would be asked about them again later in the session (3 points).

#### Task 3.2: Orientation

The participants were asked to input the current date (day, month, and year) using the digital interface (1 point).

### Attention and Working Memory (1 Point for Each Subtask, 3 Points)

#### Tasks 4 and 5: Forward and Backward Digit Span

The participants listened to and repeated increasingly long sequences of numbers, first in the same order, and then in reverse order.

#### Task 6: Visuospatial Sequencing

The participants were required to observe and replicate a sequence of changing colored circles by tapping them in the correct order.

### Executive Function (4 Points)

#### Task 2: Planning

A metro route map was displayed; participants had to analyze the map and select the specific path that contains the fewest stations between 2 designated points (1 point).

#### Task 8: Calculation

In a simulated functional task, participants were presented with a total purchase costing €38 (US $42.02; €1=US $1.1057 as of April 4, 2025) and must calculate and select all the necessary currency denominations to provide the correct change from a €50 bill (US $55.29; 3 points).

### Memory Domains (15 Points)

#### Task 7: Verbal Memory With Visual Input-Delayed Recall

This task evaluated the retrieval of the shopping list. It consists of a delayed free recall phase (Task 7.1), in which participants had to recall the original items (6 points). If free recall was incomplete, a facilitated cued recall phase (Task 7.2) was triggered to differentiate retrieval from storage deficits. A digital map of a supermarket was displayed, organized into various product sections (eg, bakery and butchery). When the participant tapped on a section, the interface displayed images of products in that category. The participant then had to identify and select the correct items from the original list (6 points). In both phases, the scoring algorithm applied a one-point penalty for each incorrect response. If a participant achieves a perfect score of 6 points in the free recall phase (Task 7.1), the facilitated cued recall phase (Task 7.2) was not administered, and the maximum score of 6 points for that phase was automatically awarded. The combined maximum for Task 7 is 12 points.

#### Task 9: Verbal Memory With Auditory Input—Delayed Recall

To assess long-term retrieval of the news broadcast, participants had to provide the 3 specific details previously encoded (3 points).

Tasks were scored based on the number of correctly recalled elements (1 point per item for Tasks 1, 3.1, 7, 9), on a dichotomous pass/fail basis (1/0 points for Tasks 2, 3.2, 4, 5, 6), or per correct denomination (1 point per item for Task 8). A total CogniPaz score was computed by summing the task scores; higher scores indicate better cognitive performance. Domain-specific scores for episodic memory and executive function were also generated. A full video demonstration in Spanish is available in Multimedia Appendix 1.

To ensure accurate and standardized quantification, CogniPaz used a deterministic rule-based scoring algorithm. For speech-based verbal recall tasks, the system transiently processed textual hypotheses returned by the native speech-recognition service, applying uppercase conversion and Spanish accent normalization. Target matching was then executed using a Levenshtein distance threshold of ≤1 to tolerate minor articulation, spelling, or speech-recognition variations. Further details on software architecture, data flow, and task-level scoring rules are provided in Multimedia Appendix 2.

A sanitized source-code artifact of the CogniPaz app is provided in Multimedia Appendix 3. This artifact is not intended to serve as a production deployment package or a fully executable clinical system. End-to-end execution requires a separately configured institutional REDCap (Vanderbilt University) backend project with matching fields, authorized private credentials, and institutionally approved deployment settings.

Upon completion, participants completed a custom-designed satisfaction questionnaire rated on a 9-point Likert scale (1=minimum; 9=maximum). This ad hoc, nonvalidated instrument covered 7 dimensions in total (general perception, ease of use, information clarity, task clarity, adequate time, task ease, and perceived utility), and was designed to capture aspects specific to a gamified, minimally supervised tablet-based assessment that are not fully covered by generic instruments. It was also evaluated for internal consistency using Cronbach alpha, which was computed among responders, alongside item-rest correlations and alpha-if-item-deleted statistics to assess the coherence of the construct.

### Conditions and Design

This was a single-center, prospective observational study of patients with cognitive complaints and HC presenting to the Cognitive Disorders Unit of La Paz University Hospital (Madrid, Spain), a tertiary referral unit specializing in cognitive neurodegenerative diseases. In accordance with nonexperimental, observational designs, the participants’ cognitive conditions were naturally observed, and no experimental manipulation was performed. This study was reported in adherence to the 2015 STARD (Standards for Reporting of Diagnostic Accuracy Studies) guidelines, with the completed checklist provided in .

### Inclusion and Exclusion

The general inclusion criteria were patients with no diagnosis of dementia presenting with cognitive complaints. The comparison group of HC consisted of companions of the patients and other healthy volunteers recruited at the hospital who were age-matched to the patient group. The exclusion criteria were (1) established dementia, defined as significant functional impairment that compromised independence in instrumental activities of daily living (Clinical Dementia Rating [CDR] global score ≥1); (2) sensory or motor deficits (eg, severe visual impairment and upper limb paresis) precluding the use of a tablet; or (3) a significant language barrier or inability to communicate proficiently.

### Participant Characteristics

Participants were classified into 3 diagnostic groups (HC, SCD, and MCI) based on a comprehensive clinical assessment integrating medical history, neurological examination, and standard neuropsychological testing. For subgroup analyses, the MCI group was further stratified into 2 phenotypes: aMCI and nonamnestic MCI. The MoCA, a widely validated instrument with established reliability and validity in the Spanish population [24], was used as the reference standard due to its sensitivity for MCI screening [25,26], and its potential value in predicting underlying AD pathology [27].

The MCI group was defined according to established clinical criteria [4,28]. Participants reported a persistent decline in cognitive capacity relative to their previous level of functioning, unrelated to an acute event, and this decline was corroborated by an informant. This was accompanied by objective impairment in at least one cognitive domain (MoCA <26, adjusted for education), while maintaining largely preserved independence in functional abilities (CDR global score of 0.5). For subgroup analyses, the aMCI subgroup was defined by predominant episodic memory impairment, whereas nonamnestic patients with MCI exhibited impairments in other cognitive domains [29].

The SCD group was defined according to the SCD-initiative framework [30]. Participants reported analogous subjective concerns regarding persistent cognitive decline but, in contrast to the MCI group, exhibited normal performance on standardized cognitive tests (MoCA ≥26, adjusted for education) and independence in functional abilities (CDR global score of 0).

HC were participants with an absence of active neurological or psychiatric disorders, no significant subjective cognitive concern, and performance within normal limits on screening (MoCA ≥26, adjusted for education).

### Sampling Procedures

Participants were recruited using a consecutive convenience sampling procedure during scheduled clinical visits between April 2025 and January 2026. During this period, all consecutive patients and their accompanying individuals attending new clinical consultations were systematically assessed for eligibility and recruited accordingly by the attending clinical and research staff to minimize selection bias. Potential candidates were provided with a detailed explanation of the study procedures prior to obtaining written informed consent. No financial compensation or other incentives were provided for participation.

### Sample Size, Power, and Precision

No formal sample size calculation was performed because no previous data were available to estimate the expected effect sizes and because of the exploratory nature of the study. Therefore, a convenience sample was used based on feasibility criteria, including the expected recruitment capacity and available resources.

### Measures and Covariates

Major demographic and clinical characteristics (including age, sex, education level, family history of AD, and MoCA scores) were collected and compared across the diagnostic groups (HC, SCD, MCI, aMCI, and nonamnestic MCI) to assess baseline differences. Structural neuroimaging (magnetic resonance imaging [MRI]), fluorodeoxyglucose positron emission tomography (FDG-PET), amyloid-PET, and cerebrospinal fluid (CSF) biomarkers (amyloid beta 42 [Aβ42], amyloid beta 42/40 ratio [Aβ42/40], phosphorylated tau [p-tau], and total tau [t-tau]) [31] were also reviewed in participants who had undergone these procedures, based on the routine clinical judgment of the attending physician. The primary investigational measures derived from the CogniPaz platform included the total cognitive score (ranging from 0 to 38) and the total administration time. Feasibility covariates included the completion rate, technical malfunctions, and the frequency of required external assistance. User satisfaction was measured using the 9-point ad hoc Likert scale questionnaire.

### Data Collection

Participants completed the assessments during a single, in-person session in a quiet room at the cognitive disorders unit. To reflect routine clinical workflow, the order of administration was fixed (MoCA followed by CogniPaz). CogniPaz was administered on a 9-inch touchscreen device (Lenovo Tab M9, Android 12), and performance metrics (including correct responses, error counts, and completion times) were automatically captured by the software. To ensure secure data gathering, the tablet front end transmitted structured assessment data securely via HTTPS POST requests to a REDCap-compatible API. The app used an automated offline contingency mechanism (Ionic Storage) to temporarily queue data locally and prevent loss during Wi-Fi interruptions in the clinical environment. The research team recorded any technical malfunctions, data loss incidents, and the frequency and nature of required external assistance. Upon completion of the cognitive tasks, participants filled out the satisfaction questionnaire.

### Quality of Measurements

To ensure standardization and enhance the quality of measurements, the research team attended an introductory training session prior to the study. To minimize interrater variability during data collection, the majority of the CogniPaz administrations were overseen by a single trained investigator. Supervision was minimal, limited strictly to technical support or task clarification to prevent administration bias.

### Masking

Routine clinical assessment and MoCA administration were performed by neurologists blinded to the participants’ CogniPaz scores. Subsequently, the self-administered, automated CogniPaz assessment was overseen by researchers who were entirely blinded to the participants’ clinical diagnosis and MoCA results.

### Data Diagnostics

A predefined protocol was established to manage data integrity prior to inferential testing. Post-data-collection exclusion criteria were set to disqualify sessions with incomplete data tracking resulting from technical disruptions or participant withdrawal. No missing data imputation methods were planned; instead, a complete-case analysis approach was prespecified for any incomplete records. In case of missing data, the Little test of missing completely at random (MCAR test) was performed to determine if missing data were missing completely at random. We also compared participants who completed the satisfaction questionnaire (“responders”) with those who did not (“nonresponders”) across demographic and clinical variables, using Welch *t* test for continuous variables and Fisher exact test for categorical variables, to assess whether noncompletion was associated with participant characteristics. Statistical outliers in continuous variables were defined using the IQR method. The normality of continuous-variable distributions was systematically evaluated using the Shapiro-Wilk test to guide the selection between parametric and nonparametric inferential strategies. Data transformations were not anticipated, as nonparametric methods were preferred to robustly handle potential skewness in neurocognitive metrics and session administration times.

### Data Analysis

Continuous variables were presented as medians and IQR if nonnormal distribution and heterogeneity of variances were observed. Between-group comparisons of continuous variables were conducted using the Kruskal-Wallis test, run separately across the 3 primary diagnostic groups (HC, SCD, and MCI) and, within MCI, across its 2 subtypes (aMCI and nonamnestic MCI). Significant global results were followed by Dunn post hoc pairwise comparisons with Bonferroni correction.

Categorical variables were analyzed based on the distribution of expected frequencies. The chi-square test was used for variables meeting the assumption of expected cell counts ≥5 (sex and education level). Fisher exact test was used for variables with sparse data violating these assumptions (family history of dementia) and for all post hoc pairwise comparisons to ensure robustness against small subgroup sizes.

Correlations between CogniPaz global scores and MoCA indices were evaluated using Spearman rank coefficients. The 95% CIs for these correlation coefficients were derived using bootstrap resampling with 5000 replications. Diagnostic accuracy of CogniPaz was examined using receiver operating characteristic (ROC) curve analyses for the following four comparisons: (1) MCI vs SCD, (2) MCI vs HC, (3) aMCI vs SCD, and (4) aMCI vs HC. For the secondary exploratory subanalysis, ROC analysis evaluated the tool’s capacity to discriminate between biologically confirmed versus nonconfirmed ND. For each analysis, the area under the curve (AUC) with 95% CIs was calculated.

The Youden Index was initially used to identify the mathematically optimal threshold for each diagnostic comparison. To optimize the tool for clinical screening, a unified provisional threshold strategy was then applied, prioritizing high sensitivity to minimize false negatives. At the provisional, hypothesis-generating, unified cutoff point, we reported sensitivity, specificity, positive predictive value (PPV), negative predictive value (NPV), and overall diagnostic accuracy, each with 95% Clopper-Pearson CI. Additionally, positive and negative likelihood ratios (LR₊ and LR-) were computed, with 95% CI derived using the log-normal method, to provide prevalence-independent measures of diagnostic performance. To characterize performance around each cutoff and support its interpretation as provisional, we calculated a threshold-performance table showing all metrics with 95% CI across a grid of candidate cutoffs (24.5, 26.5, and 28.5) for every comparison. It should be noted that MoCA scores contributed to the diagnostic group; accordingly, the primary diagnostic accuracy analyses constitute concurrent criterion validity assessment and are subject to potential incorporation bias.

To evaluate whether the tool’s performance was influenced by demographic factors, we compared unadjusted models (CogniPaz score alone) against adjusted models incorporating those variables that showed significant baseline differences between groups. Using the DeLong test for correlated ROC curves, we assessed whether the inclusion of these variables significantly improved diagnostic accuracy or if the tool’s performance remained independent of demographic characteristics. Furthermore, to quantify the specific explanatory power of the tool, we estimated the proportion of variance explained by each model using McFadden pseudo-*R*². The unique variance attributable to CogniPaz was calculated as the absolute increase in explained variance (incremental *R^2^*) observed when adding the total score to the base model containing the confounding variables.

Internal validation was performed using bootstrap resampling with 1000 iterations to assess the robustness of diagnostic estimates. Optimism-corrected AUCs, sensitivities, and specificities were derived, with corresponding 95% CIs. Leave-one-out cross-validation (LOOCV) was conducted to confirm the stability of the results.

To assess the added value of CogniPaz relative to conventional cognitive screening, we conducted a secondary analysis restricted to the biomarker-confirmed subsample with a concurrently administered MoCA. CogniPaz and MoCA discrimination for confirmed bND were compared using the DeLong test for 2 correlated ROC curves, given that both measures were obtained in the same patients. To evaluate the potential incremental value of CogniPaz beyond MoCA and age, we fitted 2 nested logistic regression models predicting confirmed status: a base model including MoCA total score and age, and a full model additionally including the CogniPaz total score. Model improvement was assessed with a likelihood-ratio test. Effect estimates for CogniPaz in the full model were reported as adjusted odds ratios (ORs) with 95% CIs.

The 4 primary ROC comparisons, the secondary bND subanalysis, and the grid of candidate cutoffs were prespecified in the analysis plan. Dunn post hoc pairwise comparisons were Bonferroni-corrected; no further correction for multiplicity was applied across the prespecified primary comparisons, and the bND subanalysis and threshold-performance grid are reported as exploratory and hypothesis-generating. Reported *P* values should be interpreted accordingly.

Statistical analyses were performed using R software (version 4.4.2; R Foundation for Statistical Computing). All tests were 2-tailed, and a *P* value <.05 was considered statistically significant.

### Ethical Considerations

The study protocol adhered to the principles of the Declaration of Helsinki and was approved by the Clinical Research Ethics Committee of La Paz University Hospital (HULP Code: PI-6915) on January 22, 2025 (Acta No. 02/2025). All participants provided written informed consent prior to enrollment. All data were deidentified at the source and stored in compliance with the General Data Protection Regulation (GDPR; EU 2016/679) and Spanish Organic Law 3/2018 on secure servers at the IdiPAZ Research Institute. No participant audio recordings or raw speech-recognition strings were stored or transmitted. No individual-level identifiable data or images of individual participants are reported. No financial compensation was provided to participants.

## Results

### Participants

A total of 195 consecutive individuals were assessed for eligibility. Of these, 111 (56.9%) were excluded, primarily due to established dementia (74/111, 66.7%) or refusal to participate (22/111, 19.8%). A total of 84 (43.1%) participants provided informed consent, met all criteria, and were formally enrolled. The test completion rate among enrolled participants was 100% (84/84), with no missing data or dropouts during the assessment. The complete participant flow, including detailed reasons for exclusion, allocation to primary diagnostic groups, and patients included in the exploratory biomarker subanalysis cohort, is depicted in Figure 2.

**Figure 2.**
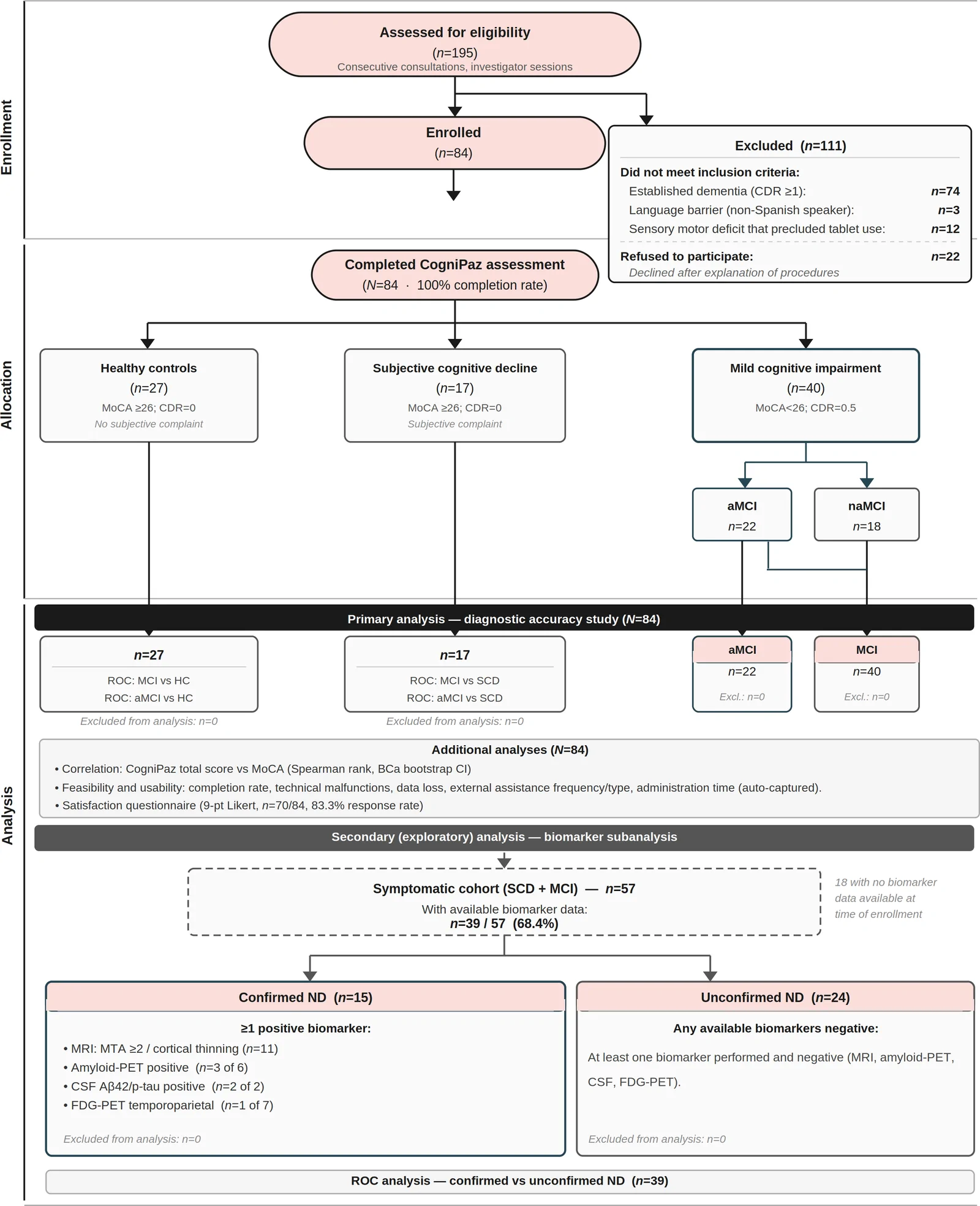
Participants flowchart, adapted from JARS-Quant guidelines. Aβ42: amyloid beta 42; aMCI: amnestic mild cognitive impairment; CDR: clinical dementia rating; CSF: cerebrospinal fluid; FDG-PET: fluorodeoxyglucose positron emission tomography; HC: healthy controls; MCI: mild cognitive impairment; MoCA: Montreal cognitive assessment; MRI: magnetic resonance imaging; MTA: medial temporal atrophy; naMCI: nonamnestic mild cognitive impairment; ND: neurodegeneration; PET: positron emission tomography; p-tau: phosphorylated tau; ROC: receiver operating characteristic; SCD: subjective cognitive decline.

### Sample Characteristics

The study cohort comprised 84 participants. Final diagnoses were 27 (32.1%) HC, 17 (20.2%) SCD, and 40 (47.6%) MCI. Within the MCI group, 22 (55.0%) participants were classified as having aMCI and 18 (45.0%) as nonamnestic MCI. Detailed demographic and clinical characteristics are summarized in Table 1.

**Table 1.** Demographic and clinical characteristics and median scores of the study population.

Characteristic	Total (n*=*84)	HC^a^ (n*=*27)	SCD^b^ (n*=*17)	MCI^c^ (n*=*40)	aMCI^d^ (n*=*22)	naMCI^e^ (n*=*18)	*P* value^f^
Age, median (IQR)	72.0 (62.5-77.0)	74.0 (59.5-77.5)	59.0 (50.0-69.0)	74.0 (69.0-78.0)	76.5 (70.8-78.8)	71.5 (68.0-74.0)	.003^g^
Women, n (%)	48 (57.1)	11 (40.7)	9 (52.9)	28 (70.0)	16 (72.7)	12 (66.7)	.12^h^
Education (college/professional training), n (%)	57 (67.9)	19 (70.4)	14 (82.4)	24 (60.0)	14 (63.6)	10 (55.6)	.37^h^
Family history of Alzheimer disease, n (%)	25 (29.8)	3 (11.1)	5 (29.4)	17 (42.5)	8 (36.4)	9 (50.0)	.02^j^
MoCA^i^ scores, median (IQR)	26.0 (23.3-27.0)	27.0 (26.0-29.0)	27.0 (27.0-28.0)	23.0 (21.0-25.0)	23.0 (21.0-24.0)	24.0 (22.0-25.0)	*<*.001^g^
CogniPaz scores, median (IQR)	28.0 (22.0-31.0)	30.0 (29.0-34.0)	31.0 (30.0-33.0)	22.5 (16.5-26.3)	19.0 (14.0-24.0)	24.0 (21.3-27.0)	*<*.001^g^

a HC: healthy control.

b SCD: subjective cognitive decline.

c MCI: mild cognitive impairment.

d aMCI: amnestic mild cognitive impairment.

e naMCI: nonamnestic mild cognitive impairment.

f *P* values correspond to the global comparison across 4 diagnostic groups: HC, SCD, aMCI, and naMCI.

g Kruskal-Wallis test.

h Chi-square test.

i MoCA: Montreal Cognitive Assessment.

j Fisher exact test.

For significant continuous variables, post hoc pairwise comparisons were performed using the Dunn test (Bonferroni-adjusted), run separately for the 3 primary groups (HC, SCD, and MCI) and for the 2 MCI subtypes (aMCI and nonamnestic MCI). Significant differences (*P*<.05) were found for age (MCI vs SCD, *P*=.02; aMCI vs SCD, *P*=.003); family history (HC vs MCI; *P*=.006); MoCA score (MCI and aMCI vs HC and SCD, all *P*<.001); and CogniPaz score (MCI and aMCI vs HC and SCD, all *P*<.001).

The median age of the cohort was 72 years. Significant age differences were observed between groups, primarily driven by SCD group being younger than those with MCI (*P*=.02) and aMCI (*P*=.003). Sex distribution and educational levels were well-balanced across all diagnostic categories. A family history of AD was significantly more prevalent in the clinical groups compared to the control group; specifically, in patients with MCI (*P*=.006).

Figure 3 illustrates the significant differences in MoCA and CogniPaz scores observed across diagnostic groups.

**Figure 3.**
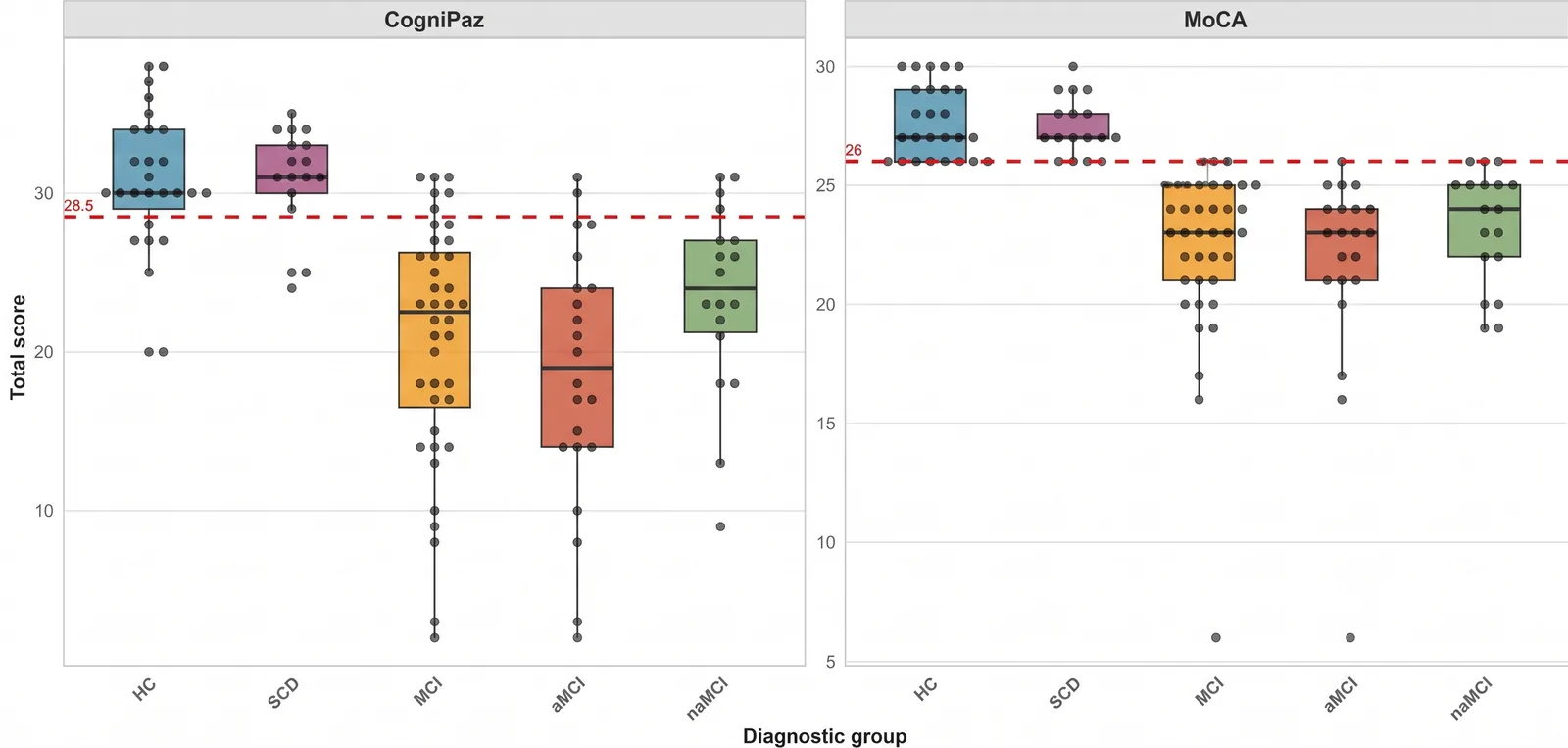
Distribution of baseline CogniPaz and Montreal Cognitive Assessment (MoCA) total scores across diagnostic groups. Boxplots show the median and IQR, and dots represent individual participants. aMCI: amnestic mild cognitive impairment; HC: healthy controls; MCI: mild cognitive impairment; naMCI: nonamnestic mild cognitive impairment; SCD: subjective cognitive decline.

MoCA scores were comparable between HC and SCD, confirming the objective normality of the SCD group on standard testing. In contrast, MCI and patients with aMCI showed significantly lower scores, with pairwise comparisons confirming significant (*P*<.001) differences compared to both HC and SCD groups. Similarly, CogniPaz total scores differed significantly between groups. While HC and SCD participants maintained preserved performance, with no significant differences between them, MCI—and particularly the aMCI subgroup—exhibited lower scores. Pairwise comparisons confirmed that patients with aMCI scored significantly lower than both HC (*P<*.001) and SCD (*P*<.001) participants, supporting the tool’s ability to discriminate objective deficits in the target population.

MoCA scores displayed a ceiling effect in the HC and SCD groups and revealed no significant differences between aMCI and nonamnestic MCI phenotypes (*P=*.23). In contrast, CogniPaz scores exhibited a wider distribution. While aMCI participants obtained numerically lower scores than nonamnestic MCI cases, this difference did not reach statistical significance (*P=*.07).

### Feasibility and Administration Time

All recruited participants completed the 9 CogniPaz tasks, achieving a 100% (84/84) task completion rate across all clinical sessions, with no indeterminate results, technical failures, software crashes, or data synchronization losses due to application malfunction reported during the study; thus, all recruited participants who completed the protocol were included in the final analysis. Regarding administration time, the total sample followed a nonnormal distribution. The median total administration time was 9.39 minutes (IQR 7.53‐11.38). Notably, when stratified by diagnosis, the individual subgroups displayed normal distributions. This difference suggests that the global nonnormality is driven by a distinct performance clustering between groups. As illustrated in Figure 4 and detailed in Table 2, both HC and SCD groups completed the assessment significantly faster than participants with MCI.

**Figure 4.**
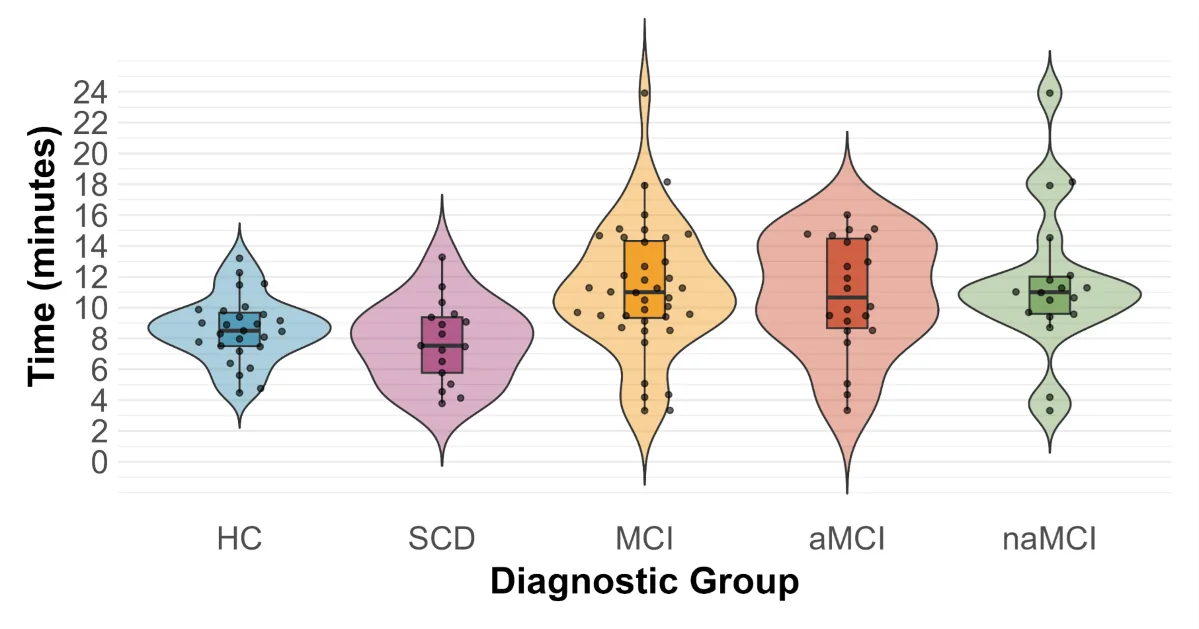
Distribution of CogniPaz administration times across diagnostic groups. Violin plots represent the distribution density of administration times (minutes), embedded boxplots show the median and interquartile range, and dots represent individual participants. aMCI: amnestic mild cognitive impairment; HC: healthy controls; MCI: mild cognitive impairment; naMCI: nonamnestic mild cognitive impairment; SCD: subjective cognitive decline.

**Table 2.** Comparison of completion times (minutes) across diagnostic groups.

Group	N	Median IQR	*P* value (vs HC)^a^	*P* value (vs SCD)^b^	*P* value (vs MCI)^c^	*P* value (vs aMCI)^d^	*P* value (vs naMCI^e^)^f^
Total	84	9.39 (7.53-11.38)	—^g^	—	—	—	—
HC	27	8.50 (7.49-9.67)	—	.99	*.02*^*h*^	.10	.06
SCD	17	7.53 (5.77-9.37)	.99	—	*.007*	*.04*	*.02*
MCI	40	10.99 (9.33-14.32)	*.02*	*.007*	—	—	—
aMCI	22	10.66 (8.67-14.47)	.10	*.04*	—	—	.99
naMCI	18	10.99 (9.60-12.01)	.06	*.02*	—	.99	—

a HC: healthy controls.

b SCD: subjective cognitive decline.

c MCI: mild cognitive impairment.

d aMCI: amnestic mild cognitive impairment.

e naMCI: nonamnestic mild cognitive impairment.

f Kruskal-Wallis test for intergroup comparisons followed by Dunn test for pairwise comparisons, run separately for the 3 primary groups (HC, SCD, and MCI) and for the 2 MCI subtypes (aMCI and nonamnestic MCI).

g Not applicable.

h Italicized values indicate statistically significant differences, *P*<.05.

### Correlation Between CogniPaz Scores and MoCA Scores

The CogniPaz total score exhibited a strong positive correlation with the MoCA total score (Spearman ρ*=*0.81, 95% CI 0.75‐0.89; *P<*.001). Furthermore, domain-specific validity was supported by high correlations between the memory-related CogniPaz tasks and the corresponding MoCA memory indices (ρ*=*0.61-0.68; *P<*.001), suggesting that the digital metrics accurately capture the targeted cognitive constructs.

### Diagnostic Accuracy

CogniPaz discriminated between MCI and HC and between MCI and SCD, with numerically higher accuracy for the amnestic MCI subgroup than for the overall MCI group. Figure 5 presents the ROC curve analyses for all diagnostic comparisons. Because aMCI is a subset of MCI, the corresponding AUCs are not statistically independent, and relative performance across these comparisons should be interpreted descriptively rather than as formally tested differences.

We further conducted a prespecified secondary exploratory subanalysis to evaluate the tool’s capacity to identify underlying bND within patients in the symptomatic cohort (SCD + MCI). Among patients with available biomarker data (n=39; 68.4% of the 57 symptomatic participants), 15 (38.5%) had confirmatory data, and 24 (61.5%) did not. The confirmed group included patients with hippocampal atrophy (medial temporal atrophy [MTA] score ≥2) and/or a reduced cortical thickness in the precuneus, entorhinal cortex, and posterior cingulate regions (11/37 MRIs performed, 29.7%), positive amyloid-PET (3/6, 50.0%), positive CSF biomarkers (2/2, 100.0%), or suggestive FDG-PET hypometabolism, defined as a temporoparietal pattern (1/7, 14.3%); the remaining cases showed nonspecific, patchy frontoparietal (2/7, 28.6%) or parietal (1/7, 14.3%) patterns, or were entirely normal (3/7, 42.9%) [32]. In this specific subsample, the tool achieved an AUC of 0.810 (95% CI 0.661‐0.958) for discriminating both categories. ROC curve analyses are presented in Figure 6.

**Figure 5.**
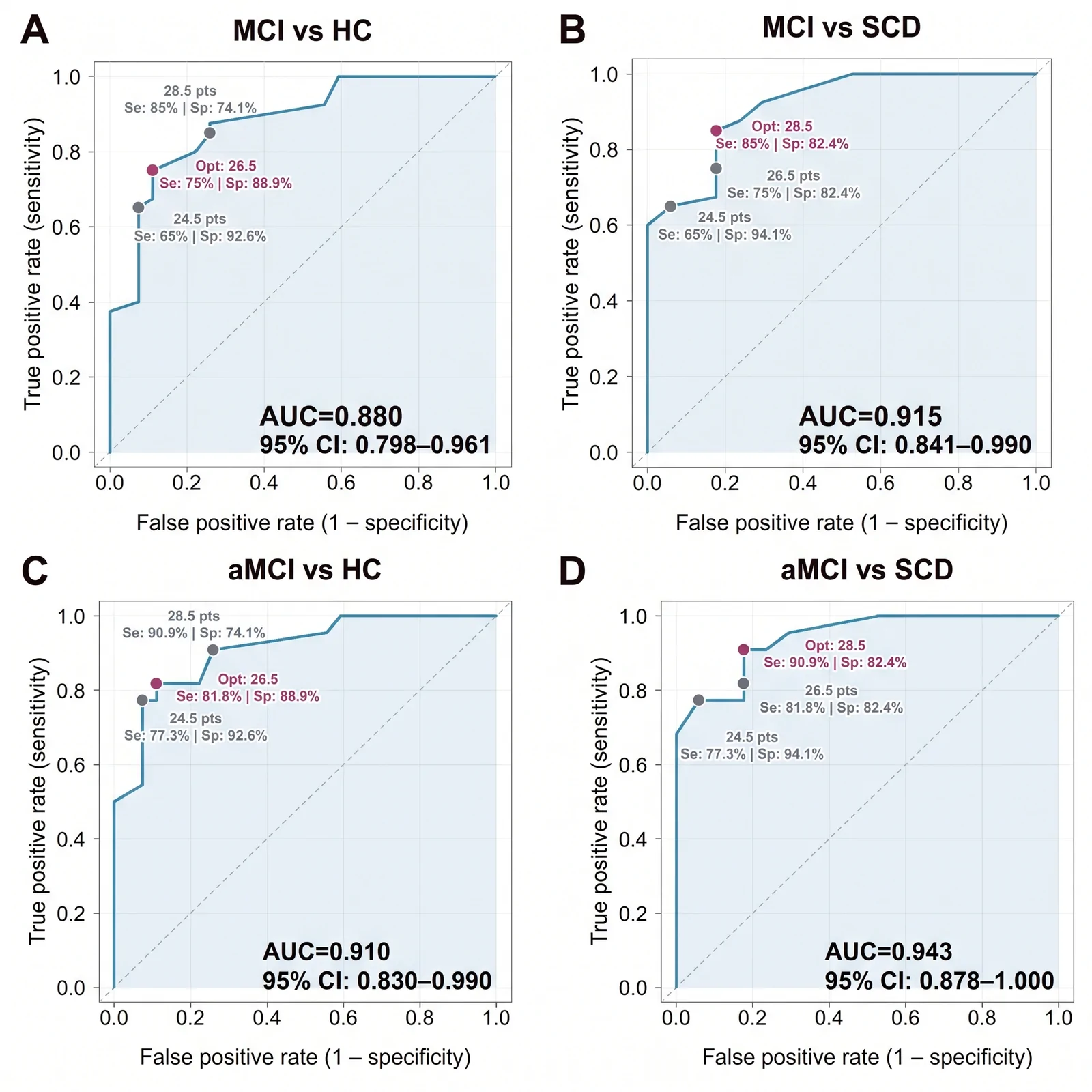
Receiver operating characteristic (ROC) curves showing the diagnostic performance of CogniPaz for identifying mild cognitive impairment (MCI) and amnestic mild cognitive impairment (aMCI) in the study population. (A) MCI versus healthy controls (HC). (B) MCI versus subjective cognitive decline (SCD). (C) aMCI versus HC. (D) aMCI versus SCD. The highlighted point in red indicates the optimal cutoff determined using the Youden index. Sensitivity and specificity at the 24.5-, 26.5-, and 28.5-point cutoffs are displayed for each comparison. The diagonal dashed line represents chance-level discrimination. aMCI: amnestic mild cognitive impairment; AUC: area under the curve; HC: healthy controls; MCI: mild cognitive impairment; Opt*:* optimal cutoff*;* Pts*:* points; Se: sensitivity; Sp: specificity.

**Figure 6.**
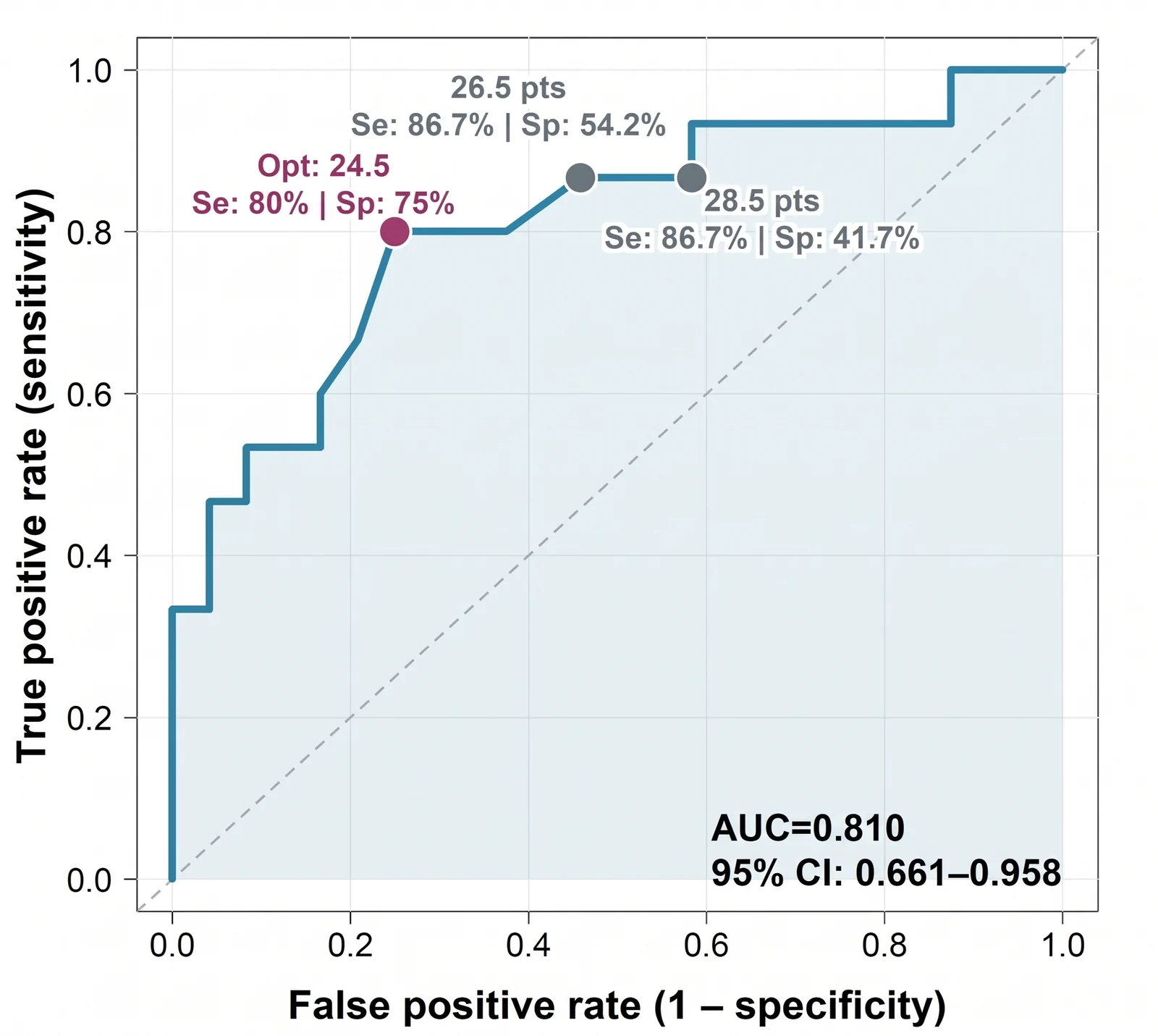
Receiver operating characteristic (ROC) curve showing the diagnostic performance of CogniPaz for identifying biologically confirmed any-cause neurodegeneration among symptomatic participants (subjective cognitive decline and mild cognitive impairment) with available biomarker data recruited from a tertiary memory clinic. The highlighted point in red indicates the optimal cutoff determined using the Youden index. The diagonal dashed line represents chance-level discrimination. Sensitivity and specificity at 24.5, 26.5, and 28.5 cutoffs are shown, highlighting the optimal threshold. AUC: area under the curve; Opt: optimal cutoff; Pts: points; Se*:* sensitivity; Sp: specificity.

To formally address the added value of CogniPaz over conventional screening of neurodegenerative etiology, we analyzed the biomarker-available subsample (n=39; 15 confirmed bND, 24 not confirmed bND). CogniPaz showed higher discrimination than MoCA for confirmed bND (AUC=0.810, 95% CI 0.661‐0.958, vs AUC=0.661, 95% CI 0.471‐0.851; DeLong *Z*=1.97; *P*=.05). In multivariable analysis, adding the CogniPaz total score to a model including MoCA and age significantly improved model fit (likelihood-ratio test: *χ*²_1_=7.46; *P*=.006), with each additional CogniPaz point associated with lower odds of confirmed bND independently of MoCA and age (adjusted OR=0.78, 95% CI 0.60‐0.94; *P*=.02).

To account for the significant differences in age identified in the baseline characteristics, we analyzed unadjusted and age-adjusted regression models. The inclusion of age did not yield a statistically significant improvement in diagnostic accuracy for any comparison, as detailed in Table 3.

**Table 3.** Comparison of diagnostic accuracy between unadjusted and age-adjusted CogniPaz models across the diagnostic subgroup comparisons.

Diagnostic comparison	Unadjusted AUC^a^ (95% CI)	Age-adjusted AUC (95% CI)
MCI^b^ vs HC^c^	0.880 (0.798‐0.961)	0.901 (0.828‐0.974)
MCI vs SCD^d^	0.915 (0.841‐0.990)	0.921 (0.847‐0.995)
aMCI^e^ vs HC	0.910 (0.830‐0.990)	0.924 (0.855‐0.994)
aMCI vs SCD	0.943 (0.878‐1.000)	0.943 (0.878‐1.000)
Confirmed ND vs Unconfirmed ND^f^	0.810 (0.661‐0.958)	0.811 (0.660‐0.961)

a AUC: area under the curve.

b MCI: mild cognitive impairment.

c HC: healthy controls.

d SCD: subjective cognitive decline.

e aMCI: amnestic mild cognitive impairment.

f ND: neurodegeneration.

The analysis of explained variance demonstrated that CogniPaz accounted for a substantial proportion of the unique variance in diagnostic classification (20.9%‐38.8%), whereas age contributed only marginal additional variance (1.4%‐2.9%). These findings suggest that the discriminative ability of the tool may reflect disease-specific cognitive impairment rather than age differences between the diagnostic groups.

To optimize clinical utility, we evaluated the most discriminative cutoff point of CogniPaz scores based on the Youden index, which identified the optimal threshold according to the balance between sensitivity and specificity depending on the comparison group. To distinguish MCI from HC, the optimal threshold was identified at 26.5 points. At this cutoff, CogniPaz demonstrated a sensitivity of 81.8% and specificity of 88.9% for aMCI vs HC (PPV: 85.7%, NPV: 85.7%) and a sensitivity of 75.0% and specificity of 88.9% for MCI vs HC (PPV: 90.9%, NPV: 70.6%). Conversely, to distinguish MCI from SCD, the optimal threshold shifted to 28.5 points, as shown in Figure 5.

Since the primary objective of CogniPaz is to serve as an effective screening instrument, we prioritized sensitivity to minimize the risk of missing cases by establishing a provisional unified cutoff of 28.5 points (scores below this threshold indicate cognitive impairment). The shift from 26.5 to 28.5 resulted in an improvement in sensitivity for both aMCI vs HC (from 81.8% to 90.9%) and MCI vs HC (from 75.0% to 85.0%). This gain in sensitivity came with a trade-off in specificity, which decreased from 88.9% to 74.1% for both MCI vs HC and aMCI vs HC, as depicted in Figure 7.

In the subanalysis of bND, although the 28.5-point cutoff maintained good sensitivity (86.7%), this came at the cost of lower specificity (41.7%). The optimal 24.5 threshold provided a more balanced performance for identifying bND, increasing specificity to 75.0% and overall accuracy to 76.9% vs 59.0% at the 28.5 cutoff. The 95% CIs for all metrics at every candidate cutoff are provided in Table S1 in Multimedia Appendix 4.

At this level, 81.0% of the total cohort (68/84 participants) was correctly classified. Table 4 presents the final performance metrics at the provisional 28.5-point cutoff for the clinical comparisons and, separately, at the 24.5-point cutoff for the exploratory bND subanalysis; the complete threshold-performance table across all candidate cutoffs (24.5, 26.5, and 28.5), with 95% CIs for every metric, is provided in Table S1 in Multimedia Appendix 4.

**Figure 7.**
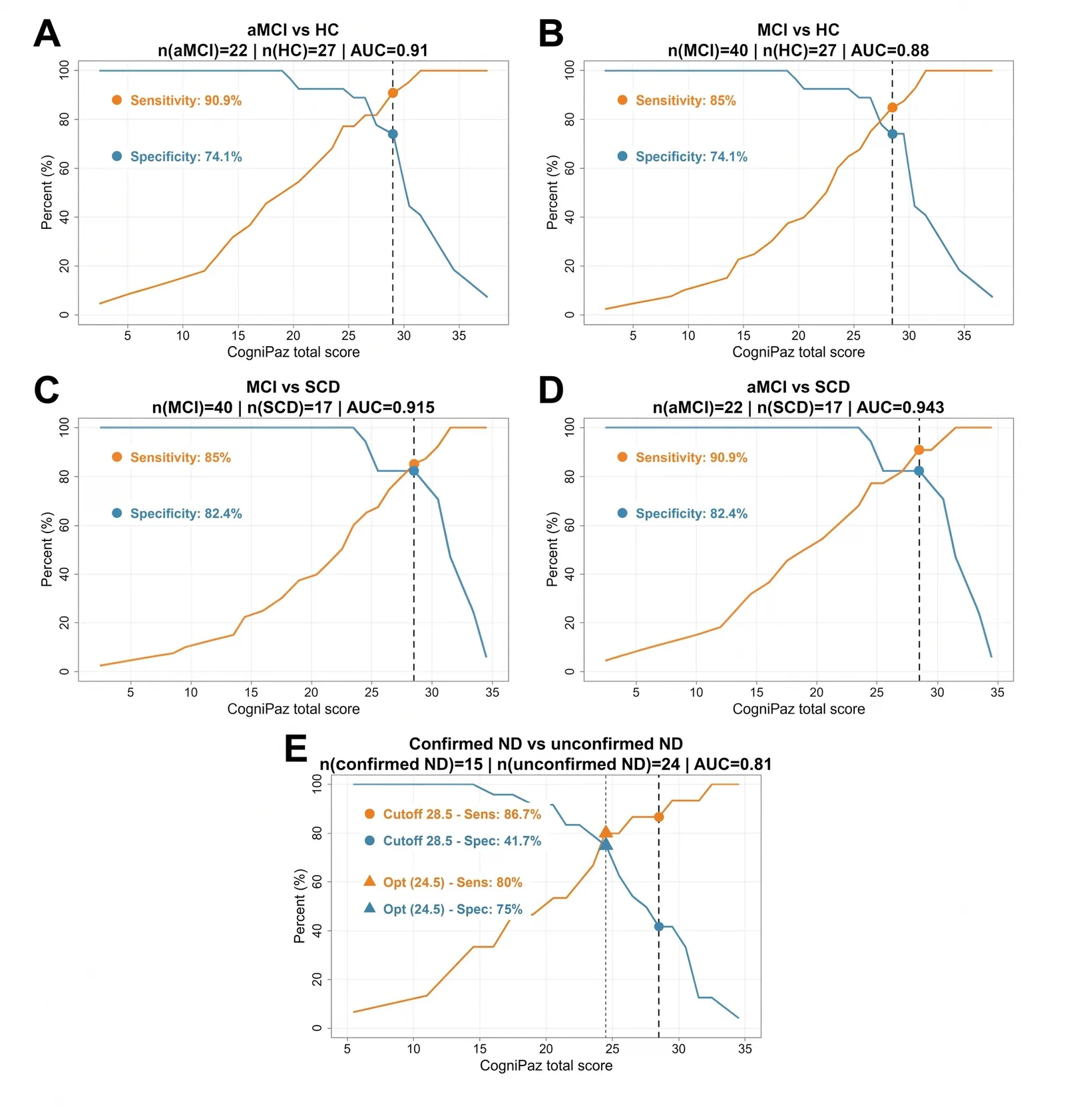
Sensitivity and specificity of the CogniPaz digital cognitive screening tool across the evaluated diagnostic comparisons. (A) amnestic mild cognitive impairment (aMCI) versus healthy controls (HC). (B) mild cognitive impairment (MCI) versus HC. (C) MCI versus subjective cognitive decline (SCD). (D) aMCI versus SCD. (E) Biologically confirmed versus unconfirmed neurodegeneration. Orange and blue curves represent sensitivity and specificity, respectively, across the range of CogniPaz total scores. Dashed vertical lines indicate the provisional 28.5-point cutoff; in panel E, the dotted vertical line indicates the alternative 24.5-point cutoff. aMCI: amnestic mild cognitive impairment; HC: healthy controls; MCI: mild cognitive impairment; ND*:* neurodegeneration; SCD: subjective cognitive decline.

**Table 4.** Diagnostic performance and classification accuracy of the CogniPaz tool across clinical comparisons at the 28.5 cutoff and at the 24.5 cutoff for biologically confirmed any-cause neurodegeneration.

Diagnostic comparison	TP^a^	TN^b^	FP^c^	FN^d^	Sens^e^ (95% CI)	Spec^f^ (95% CI)	PPV^g^ (95% CI)	NPV^h^ (95% CI)	LR^i^+ (95% CI)	LR-^j^ (95% CI)	Accuracy (95% CI)
MCI^k^ vs HC^l^	34	20	7	6	85.0 (70.2‐94.3)	74.1 (53.7‐88.9)	82.9 (67.9‐92.8)	76.9 (56.4‐91.0)	3.28 (1.71‐6.28)	0.20 (0.09‐0.44)	80.6 (69.1‐89.2)
aMCI^m^ vs HC	20	20	7	2	90.9 (70.8‐98.9)	74.1 (53.7‐88.9)	74.1 (53.7‐88.9)	90.9 (70.8‐98.9)	3.51 (1.83‐6.72)	0.12 (0.03‐0.47)	81.6 (68.0‐91.2)
MCI vs SCD^n^	34	14	3	6	85.0 (70.2‐94.3)	82.4 (56.6‐96.2)	91.9 (78.1‐98.3)	70.0 (45.7‐88.1)	4.82 (1.71‐13.56)	0.18 (0.08‐0.39)	84.2 (72.1‐92.5)
aMCI vs SCD	20	14	3	2	90.9 (70.8‐98.9)	82.4 (56.6‐96.2)	87.0 (66.4‐97.2)	87.5 (61.7‐98.4)	5.15 (1.83‐14.51)	0.11 (0.03‐0.42)	87.2 (72.6‐95.7)
Confirmed ND^o^ vs unconfirmed ND (28.5 cutoff)	13	10	14	2	86.7 (59.5‐98.3)	41.7 (22.1‐63.4)	48.1 (28.7‐68.1)	83.3 (51.6‐97.9)	1.49 (1.00‐2.20)	0.32 (0.08‐1.26)	59.0 (42.1‐74.4)
Confirmed ND vs unconfirmed ND (24.5 cutoff)	12	18	6	3	80.0 (51.9‐95.7)	75.0 (53.3‐90.2)	66.7 (41.0‐86.7)	85.7 (63.7‐97.0)	3.20 (1.53‐6.69)	0.27 (0.09‐0.75)	76.9 (60.7‐88.9)

a TP: true positive.

b TN: true negative.

c FP: false positive.

d FN: false negative.

e Sens: sensitivity.

f Spec: specificity.

g PPV: positive predictive value.

h NPV: negative predictive value.

i LR⁺: positive likelihood ratio.

j LR⁻: negative likelihood ratio.

k MCI: mild cognitive impairment.

l HC: healthy controls.

m aMCI: amnestic mild cognitive impairment.

n SCD: subjective cognitive decline.

o ND: neurodegeneration.

Internal validation using 1000 bootstrap resamples confirmed the robustness of these diagnostic estimates. The optimism-corrected AUCs were virtually identical to the apparent values for all comparisons (eg, aMCI vs SCD: apparent AUC=0.943 vs optimism-corrected AUC=0.942; 95% CI 0.866‐0.989). Sensitivity and specificity at the 28.5 cutoff remained stable after correction (aMCI vs SCD: sensitivity 90.9% vs 90.7%; specificity 82.4% vs 82.5%), indicating minimal overfitting and supporting the generalizability of the findings within the target population (see Table S2 in Multimedia Appendix 5 for complete bootstrap results). LOOCV yielded consistent results, further reinforcing the stability of the model.

### Usability and Satisfaction

Of the 84 participants, 70 (83.3%) completed the usability questionnaire and 14 (16.7%) did not. Responders and nonresponders did not differ significantly in age, sex, diagnostic group, or MoCA score. Nonresponders more frequently required assistance with the tablet during testing (69.2% vs 41.2%; *P=*.08). Nonresponders also had a significantly higher prevalence of first-degree family history of AD (57.1% vs 25.4%; *P=*.03). The Little MCAR test applied to the 7 questionnaire items alone did not reject the null hypothesis of data missing completely at random (*χ*²_6_=8.86; *P=*.18); however, when age, sex, family history, need for tablet assistance, and MoCA score were included, the test rejected MCAR (*χ*²_49_=69.77; *P=*.03), in line with the association between nonresponse and family history noted above. Missingness in this questionnaire was therefore better characterized as missing at random (MAR), conditional on observed covariates, rather than missing completely at random. The 7-item questionnaire showed good internal consistency (Cronbach α=0.84, 95% CI 0.78‐0.89), with all item-rest correlations ≥0.46 and no item improving reliability if removed.

Overall usability ratings were high, with a median score of 8.43 (IQR 7.86‐8.57) on a 9-point scale. Item-level analysis indicated high ratings across domains, with median scores of 9.0 for “Perceived Utility,” “Information Clarity,” “Task Clarity,” and “Adequate Time“ (all IQR 8.0‐9.0) across all diagnostic groups. “Tasks Ease“ yielded the lowest median score (median 8.0, IQR 6.25‐9.0), as illustrated in Figure 8.

**Figure 8.**
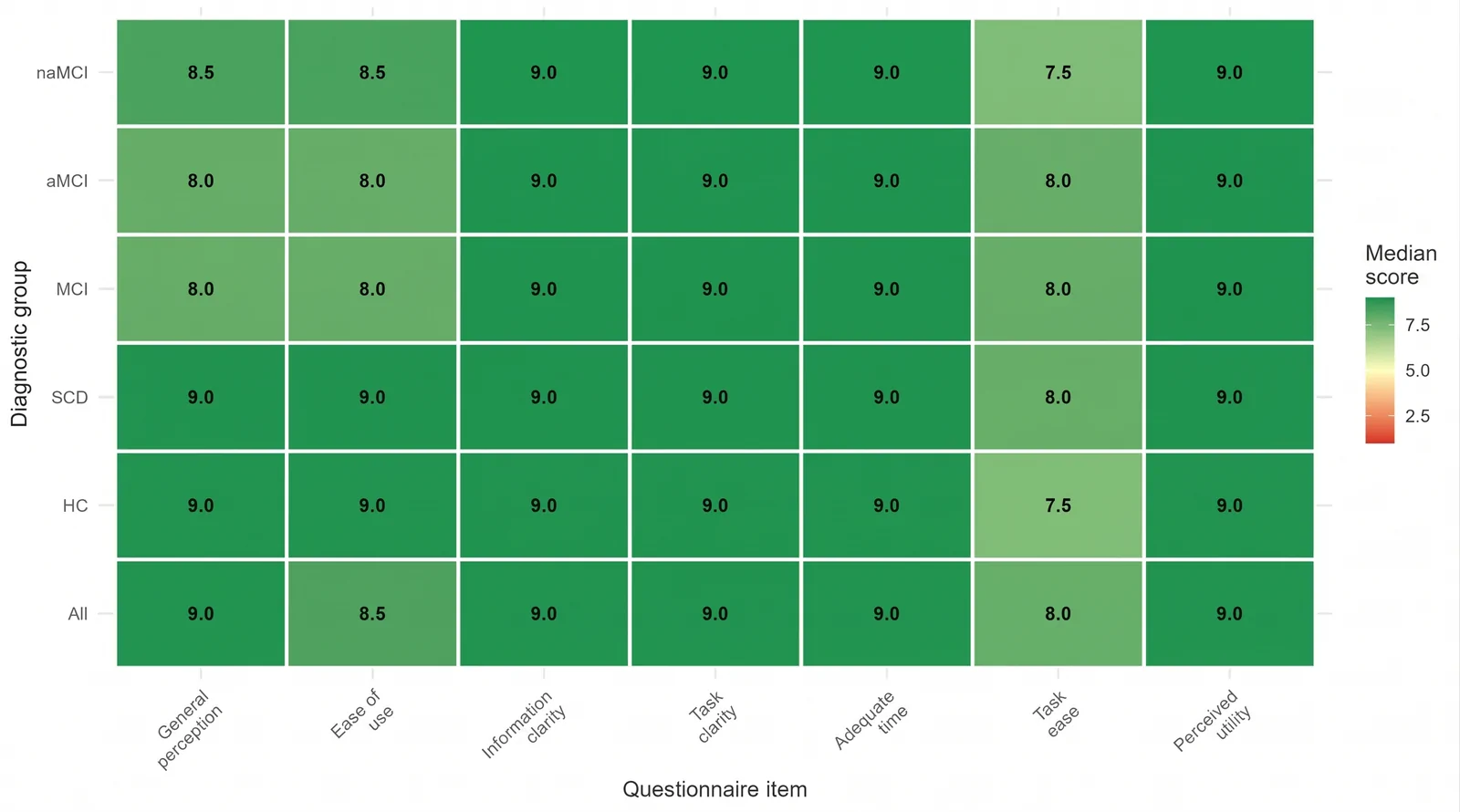
Median satisfaction scores by diagnostic group and item. Heatmap colors and cell values represent the median score for each questionnaire item on a 1‐9 scale. “ALL” indicates the overall study population. aMCI: amnestic mild cognitive impairment; HC: healthy controls; MCI: mild cognitive impairment; naMCI: nonamnestic mild cognitive impairment; SCD: subjective cognitive decline.

While HC and SCD participants reported high and homogeneous satisfaction scores (HC: median 8.50, IQR 8.00-8.61; SCD: median 8.43, IQR 8.29-8.57), the MCI subgroups exhibited wider IQR, reflecting a much broader dispersion in user experience. Specifically, the aMCI subgroup showed a median satisfaction score of 8.17, but with a wider dispersion (IQR 7.29-8.57) compared to the groups with no cognitive impairment; similarly, the nonamnestic MCI phenotype presented a median satisfaction score of 8.00 (IQR 7.61-8.61). Statistical analysis confirmed no significant correlation between age and satisfaction scores (Spearman ρ*=*–0.16; *P=*.20).

Moreover, the use of the tool required minimal supervision: 54.3% of the cohort completed the protocol with zero assistance, while the remaining participants required only minor guidance (limited to 1‐2 interventions), which primarily involved assistance with microphone usage or clarification of the task instructions. However, the rate of independent use varied significantly by group: 87.5% for SCD, 69.2% for HC, and only 30.8% for the MCI group (ie, most patients required some support). A significant negative correlation was found between the frequency of assistance and total CogniPaz scores (*r=*–0.505; *P<*.001). Participants requiring guidance achieved significantly lower global scores (mean 21.51, SD 8.44) compared to those who were fully autonomous (mean 29.48, SD 5.17; *P<*.001). No systematic adverse reactions (eg, frustration and fatigue) were reported.

## Discussion

### Principal Findings

In this study, CogniPaz showed good, concurrent capacity to differentiate MCI, particularly the amnestic phenotype, from HC and SCD. It also exhibited a strong correlation with MoCA and proved feasible for routine clinical use, achieving good user satisfaction and requiring minimal supervision. Finally, the tool suggested a preliminary capacity to identify underlying bND among symptomatic patients.

### Related Work

Different digital tools aimed at assessing cognitive disorders are available, with varying degrees of validation [5,6,15]. Established computerized batteries, such as the Cogstate Brief Battery (CBB), which focuses on processing speed and learning, have demonstrated moderate accuracy for discriminating MCI from cognitively unimpaired participants (AUC≈0.75), achieving improved performance (AUC up to 0.93) only when biomarker-defined prodromal AD is considered [33]. Similarly, BrainCheck (BrainCheck, Inc), a multidomain battery, has reported sensitivities and specificities between 77% and 88% for MCI and dementia classification [34], while Philips IntelliSpace Cognition (ISC) offers tablet-based versions of conventional tests, with evidence of measurement equivalence [35].

Beyond the mere digitization of traditional assessments, recent diagnostic approaches have incorporated automated analysis of process-based metrics and machine learning algorithms to refine early detection [5,6,15,16]. A linear support vector model using digital metrics from the “What was where?” Oxford Memory Task (OMT) has shown high diagnostic accuracy across the AD continuum. This tablet-based tool achieved an AUC of 0.82 in discriminating between HC and those with SCD and an even higher AUC of 0.92 for distinguishing SCD from MCI. While its performance in classifying MCI versus AD dementia was lower (AUC=0.75), the tool’s identification accuracy was a top predictor of hippocampal atrophy, performing as effectively as standard neuropsychological screenings [36]. Mobile-based solutions like the Digital Assessment of Cognitive Impairment (DACI) have achieved AUCs around 0.87‐0.90 in identifying cognitively impaired older adults, with administration times under 5 minutes [37], while ALTOIDA-iADL (Altoida Inc), using augmented reality to simulate instrumental activities of daily living, demonstrated robust diagnostic accuracy (AUC≈0.78) in distinguishing nondegenerative MCI from prodromal AD in patients characterized by cerebrospinal fluid biomarkers [38]. Other emerging digital solutions include remote episodic memory composites, unsupervised paired-associate learning tasks, and app-based screeners such as digiDEM-SCREEN (digiDEM Bayern project, Friedrich-Alexander-Universität Erlangen-Nürnberg) [9,39].

Expanding into self-administered and remote solutions presents both opportunities and trade-offs regarding diagnostic certainty. The Geras Solutions Cognitive Test (GSCT) proved viable for unsupervised screening (AUC=0.740 for MCI vs SCD, AUC=0.960 for detecting dementia) [40,41], while the remote, unsupervised Cambridge Neuropsychological Test Automated Battery (CANTAB) Paired Associates Learning (PAL) demonstrated feasibility in the Brain Health Registry, despite modest accuracy (AUC=0.66‐0.68 for self-reported MCI) compared to supervised versions [42]. In parallel, the digitalization of the “gold standard” MoCA has evolved from the eMoCA (which established measurement equivalence with the paper version; concordance correlation coefficient=0.84; mean difference <2 points [43]) to self-administered solutions like MoCA Solo [44] and MoCA XpressO. However, recent validation of XpressO highlights the complexity of unassisted screening: while effective at extremes, it yielded an “indeterminate range“ (scores 43‐71) where diagnostic certainty is reduced, achieving good sensitivity and specificity only outside this broad window [45].

Finally, this digital transformation is exemplified by large-scale initiatives aiming to democratize early detection. Prominent examples include SCAP-AD (Cognitive Screening and Personalized Approach for Alzheimer’s and other Dementias, Fundación CIEN) [46], a research-driven project focused on developing screening algorithms for AD in the general population, and industry collaborations, such as “The Mind Guardian” (Samsung) [47], which promotes accessibility and cognitive monitoring.

From a digital health and implementation science perspective, CogniPaz should be considered a minimally supervised mHealth cognitive-assessment device rather than a standalone diagnostic algorithm. As highlighted in recent mHealth literature, the successful adoption of digital systems relies heavily on human-centered design, proof-of-concept validation, and thorough usability assessment [23]. The novelty of CogniPaz lies in applying these core mHealth design principles to a screening environment. Consequently, its primary value proposition is not based on diagnostic superiority alone but on how it overcomes practical implementation barriers. By integrating a multi-domain evaluation into an ecological and gamified format, the brief assessment can be entirely self-administered. As demonstrated in our feasibility analysis, these characteristics allow for minimal oversight from nonspecialist personnel, such as general nurses or accompanying family members, offering a highly scalable triage solution that may help mitigate administrative bottlenecks in real-world clinical settings.

Beyond these tools, CogniPaz offers several advantages based on its unique clinical and design features. While most published digital tools primarily contrast MCI or dementia with normal cognition or examine biomarker-defined AD stages [5,6,13-15,17,20,48], CogniPaz demonstrates good accuracy in distinguishing aMCI—the primary target for early interventions—from SCD, thus allowing identification of clinically relevant early cognitive deficits. Early cognitive deficits represent a diagnostic challenge frequently encountered in specialized settings but are less often studied in digital validation. In this regard, CogniPaz displays good accuracy, identifying a provisional threshold that maximizes sensitivity. While digital adaptations of traditional scales like eMoCA or MoCA XpressO can be hampered by broad ’indeterminate ranges’ where diagnostic confidence drops [43,45], CogniPaz provides a provisional threshold for classification. With its good sensitivity and specificity for detecting aMCI versus SCD, it makes it a more practical instrument for rapid clinical triage. Specifically, the tool’s likelihood ratios indicate that, while a positive result provides moderate evidence for confirming aMCI, a negative result offers evidence for ruling it out—a particularly valuable combination for clinical decision-making in memory clinic settings. Our findings suggest that CogniPaz may assist in establishing a clinical suspicion of bND versus nonneurodegenerative etiologies, a capability rarely available in brief, digital screening tools. In particular, its preliminary performance in identifying bND etiology suggests a high rule-out capacity that could be useful in clinical triage. Within this biomarker-confirmed subsample, CogniPaz showed higher point-estimate discrimination than the MoCA, and its addition to a model already containing MoCA score and age significantly improved model fit, a pattern that may suggest potential incremental value beyond conventional screening.

Our data indicate that CogniPaz scores are not biased by age, a limitation of many computerized batteries—such as CBB, CANTAB, BrainCheck, and Philips ISC—that require the use of complex, age-stratified normative databases to distinguish pathology from normal aging [6,17]. Since the tool’s discriminative power appears to be driven by disease-specific deficits rather than age-related slowing, it allows for direct clinical interpretation without the need for lookup tables. This makes CogniPaz inherently more practical for rapid screening in diverse older adult populations.

Compared to other digital tools that emphasize episodic memory or processing speed [5,6,13-15,17,20,48], CogniPaz integrates memory and executive tasks for simultaneous assessment of memory and executive functions. Its strong correlation with the MoCA suggests that it effectively captures overall cognitive functioning, while its multidomain approach may enhance sensitivity to the early dysexecutive changes often observed in prodromal neurodegenerative syndromes. Notably, CogniPaz provides a more granular evaluation of episodic memory than traditional screens; for instance, while the MoCA uses a 5-word recall task, CogniPaz uses a 6-word list, presented through dual modalities—verbal memory with visual and auditory input. Despite this increased depth and the inclusion of multimodal assessments, the tool remains efficient. With a brief administration time, CogniPaz is competitive with existing digital batteries and shorter than many paper-based screens, successfully balancing diagnostic precision with the brevity required for routine clinical use.

The tool’s gamified and ecologically oriented design ensures high user acceptance, a critical requirement for digital health interventions highlighted by previous research [5,6,13-15,17,20,48], especially for older adults and individuals with lower educational levels. In our sample, user satisfaction was high across all domains, and no major usability issues were reported. While some participants acknowledged the inherent difficulty of certain tasks—likely reflecting the subjective challenge associated with objective performance deficits in cognitively impaired individuals—high ratings in “perceived utility” across all diagnostic groups indicate that patients recognized and valued the tool’s clinical relevance. Such intuitive and engaging interfaces may mitigate test anxiety and promote long-term engagement, thereby facilitating repeated or longitudinal assessments.

### Limitations

Several limitations must be acknowledged. The sample size, particularly for the SCD group, was modest, and confidence intervals around AUC estimates (as well as around LRs) remain relatively wide. This low level of precision reflects the exploratory nature of the study of a novel tool with a modest sample size, broadly comparable to other early-stage digital cognitive assessment-tool validation studies [19,34,39,44]. Nevertheless, the robustness of these preliminary findings is supported by the study’s prospective design and its execution within a real-world clinical setting. That said, larger, multicenter, prospective validation studies are necessary to confirm these findings and ensure their generalizability across more diverse populations and clinical environments.

Consistent with real-world clinical demographics, MCI participants were significantly older than SCD participants. While age differences can theoretically confound cognitive performance, statistical comparison of ROC curves demonstrated that adding age to the predictive models did not affect accuracy for any comparison. Furthermore, analysis of explained variance revealed that CogniPaz scores accounted for most of the predictive power, whereas age contributed marginally. This supports the tool’s validity as a disease-detection instrument, independent of demographic confounders.

Regarding clinical characterization, we acknowledge a lack of systematic quantification of potential confounders. Unlike explanatory trials with strict exclusionary criteria, our study did not exclude patients with common comorbidities, such as depressive symptoms, vascular pathology, or sensory deficits, nor did we grade their severity using standardized scales. While this decision preserves the ecological validity of a “real-world” clinical cohort, it limits our ability to determine the extent to which these nonneurodegenerative factors—rather than incipient neurodegeneration—may have contributed to performance variability, particularly in the SCD group.

Biomarker data (eg, amyloid/tau PET and CSF markers) were not systematically available for the entire cohort, as this was not the primary aim of the study. This limitation reflects the real-world setting of a tertiary memory clinic, where many participants were patients with mild symptoms, attending their first specialist consultation, at which point invasive or expensive confirmatory tests had not yet been performed. Consequently, our preliminary biological validation represents an exploratory subanalysis. While we identified a subset of bND cases with good sensitivity, the small sample size limits the statistical power and generalizability of these findings. This may have resulted in an underestimation of the tool’s diagnostic accuracy in this group, as the “unconfirmed” group may include individuals with latent neurodegenerative pathology who had not yet undergone definitive testing. These preliminary findings warrant future studies with systematic biomarker profiling before establishing CogniPaz as a digital proxy for bND and should be regarded as hypothesis-generating rather than conclusive evidence of added value.

The composite bND outcome combined heterogeneous biomarker modalities (MRI, amyloid-PET, CSF, and FDG-PET) that were obtained through routine clinical judgment rather than a uniform protocol. Participants were not verified with the same set of tests, and the modalities differ in their sensitivity and specificity for detecting neurodegeneration, so the “confirmed” and “unconfirmed” labels may not represent a fully consistent construct across participants.

The usability questionnaire used was an ad hoc, nonvalidated instrument rather than a standardized tool integrating both researcher and patient perspectives, allowing a more comparable, reproducible appraisal [49]. The 7-item scale nonetheless showed good internal consistency, supporting its interpretation as a coherent construct within this study. Furthermore, a minority of participants did not complete the questionnaire. While responders and nonresponders did not differ significantly on most measured demographic or cognitive variables, the Little MCAR test formally rejected the assumption of data missing completely at random, indicating that nonresponse was systematically related to observed participant characteristics rather than occurring purely by chance. This reinforces the need for caution when interpreting aggregate usability scores. Future iterations of CogniPaz should incorporate a validated usability instrument administered with strategies to minimize missing data, particularly among participants at higher risk of non-response.

We also did not include longitudinal follow-up in this initial, preliminary validation. Consequently, we were unable to estimate the test-retest reliability of the CogniPaz battery, and we cannot yet determine the prognostic value of CogniPaz scores for predicting conversion from SCD to MCI or from MCI to dementia. Longitudinal digital assessments, including remote or home-based administration to formally assess test-retest reliability and clinical trajectories, could be considered for future work.

We acknowledge a potential incorporation bias, as the MoCA test was utilized as one of the operational criteria for defining the aMCI group. Theoretically, validating a new tool against a standard that forms part of the diagnosis could inflate diagnostic accuracy estimates. However, the clinical classification of aMCI was not determined by MoCA cutoffs alone but through a comprehensive clinical adjudication process involving the CDR, functional history, and expert neurological consensus. Therefore, the strong relationship observed between CogniPaz and MoCA should be interpreted as evidence of solid concurrent validity against the current standard of care, demonstrating that our digital tool successfully captures the same cognitive constructs as traditional, paper-based screening, without being solely a statistical artifact of the inclusion criteria. Beyond incorporation bias in the statistical sense, the index test and the reference standard tap partially overlapping cognitive constructs by design: both CogniPaz and the MoCA assess verbal word-list recall, and aMCI is itself defined by predominant episodic memory impairment. Part of the observed correlation and of the discriminative accuracy for aMCI may therefore reflect shared method rather than fully independent validity.

The optimal cutoff score of 28.5 points was derived and tested within the same cohort, introducing a risk of overfitting. Although internal validation using bootstrap resampling suggested stability of the estimates, this does not replace external validation. Consequently, this threshold should be considered provisional and hypothesis-generating, and its generalizability to other populations and clinical settings remains unproven. The enrollment design presents an inherent risk of spectrum bias. Because this study was conducted within a tertiary memory clinic, the clinical spectrum of our cohort may not fully mirror that of general community or primary care populations where digital screening tools are frequently deployed. Using a convenience sample of accompanying individuals as HC could artificially amplify the contrast between typical aging and cognitive impairment. The relationship of each control to a study patient (eg, spouse, other relative, or unrelated volunteer) was not systematically recorded, so shared lifestyle, socioeconomic, or genetic factors within this group could not be assessed in more detail. This may be partly mitigated by the study’s focus on differentiating subtle cognitive states, such as aMCI from SCD, rather than extreme phenotypes. Independent confirmation of both the cutoff and the tool’s generalizability in larger, multicenter cohorts spanning primary care and community settings is therefore an essential prerequisite before clinical implementation can be recommended.

We were unable to calculate the word error rate (WER) or perform a post hoc inter-rater agreement analysis between the automated system and manual transcriptions. To prioritize participant privacy and satisfy regulatory constraints, the application was architected to fully discard all audio recordings and raw speech-recognition text strings immediately after task execution. While this design prevents retrospective acoustic or linguistic auditing, the platform demonstrated reliable technical feasibility during clinical evaluation, with zero technical failures or data losses. Potential automatic speech recognition (ASR) errors were mitigated in real time through live user-interface safeguards (including real-time word visualization, the ability to manually delete misrecognized items, and the option to retry the task), requiring only minimal examiner supervision. Nevertheless, future iterations of the platform should incorporate prospective, item-level logging alongside parallel manual validation protocols to formally quantify ASR diagnostic accuracy.

Finally, this study focused on supervised administration in a specialized hospital setting; we did not examine unsupervised or remote use, which is increasingly relevant for population-level screening and monitoring [17]. In our study, the need for external support was not attributable to interface complexity but was strongly driven by the participants’ cognitive status. Collectively, these findings suggest that requests for assistance reflect the executive and memory deficits inherent to the MCI condition, rather than limitations in the clarity or usability of the CogniPaz platform. Additional work is needed to determine whether the gamified interface of CogniPaz translates into consistent performance under remote conditions and how best to adapt or simplify tasks, if needed.

### Future Directions

Future research on CogniPaz should pursue several lines. First, larger normative datasets stratified by age, education, and culture are necessary to refine cutoffs and ensure fair interpretation across populations. Furthermore, longitudinal validation through repeated assessments in SCD and MCI cohorts will help determine whether CogniPaz scores and trajectories predict clinical progression or stability. Integrating these digital metrics with multimodal biomarkers—including neuroimaging, fluid and serum markers, and genetic risk profiles—may further enhance early detection and allow for the derivation of composite risk scores. Finally, evaluating CogniPaz in nonspecialized settings is a critical next step to establish its utility as a first-line triage tool; to this end, future validation studies will be extended to the primary care settings within the La Paz University Hospital health area to assess the tool’s effectiveness and feasibility as a scalable screening instrument in real-world community practice.

### Conclusions

CogniPaz is an innovative mHealth tool for early screening of cognitive impairment, designed as a multidomain, gamified, and ecologically oriented framework that includes multimodal memory and executive tasks. It differs from traditional screening tools by offering a brief, self-administered format that requires minimal supervision, makes use of the inherent advantages of digital platforms, and targets the clinically demanding aMCI-versus-SCD boundary. In doing so, it brings to the field a practical, user-friendly instrument capable of addressing shortcomings of classical screening tools. CogniPaz shows good concurrent capacity to distinguish MCI, particularly the amnestic phenotype, from SCD and HC, alongside high user acceptance. Additionally, it showed preliminary, hypothesis-generating capacity to identify bND. These results require further external, multicenter, longitudinal validation to confirm the diagnostic capacity and prognostic value of the tool in daily practice. If validated, this digital approach may provide more efficient pathways for cognitive assessment in primary care and earlier triage of patients with cognitive decline to specialized care.

## Supplementary material

10.2196/99688 Multimedia Appendix 1 Video demonstration of the CogniPaz tool in use (Spanish).

10.2196/99688 Multimedia Appendix 2 Technical software documentation, architecture diagrams, data-flow descriptions, and scoring workflows.

10.2196/99688 Multimedia Appendix 3 Sanitized source-code artifact of the CogniPaz application.

10.2196/99688 Multimedia Appendix 4 Table S1, complete threshold-performance data across all candidate cutoffs (24.5, 26.5, 28.5) for every comparison, including all metrics with 95% CI.

10.2196/99688 Multimedia Appendix 5 Table S2, complete bootstrap validation results.

10.2196/99688 Checklist 1 Completed STARD 2015 checklist.

## Abbreviations

AD: Alzheimer disease
aMCI: amnestic mild cognitive impairment
ASR: automatic speech recognition
AUC: area under the curve
Aβ42: amyloid beta 42
Aβ42/40: amyloid beta 42/40 ratio
bND: biologically confirmed any-cause neurodegeneration
CANTAB: Cambridge Neuropsychological Test Automated Battery
CBB: Cogstate Brief Battery
CDR: Clinical Dementia Rating
CSF: cerebrospinal fluid
DACI: Digital Assessment of Cognitive Impairment
FDG-PET: fluorodeoxyglucose positron emission tomography
GDPR: General Data Protection Regulation
GSCT: Geras Solutions Cognitive Test
HC: healthy controls
ISC: IntelliSpace Cognition
LOOCV: leave-one-out cross-validation
LR⁺: positive likelihood ratio
LR⁻: negative likelihood ratio
MAR: missing at random
MCAR: missing completely at random
MCI: mild cognitive impairment
mHealth: mobile health
MMSE: Mini-Mental State Examination
MoCA: Montreal Cognitive Assessment
MRI: magnetic resonance imaging
MTA: medial temporal atrophy
NPV: negative predictive value
OMT: Oxford Memory Task
OR: odds ratio
p-tau: phosphorylated tau
PAL: Paired Associates Learning
PET: positron emission tomography
PPV: positive predictive value
ROC: receiver operating characteristic
SCAP-AD: Cognitive Screening and Personalized Approach for Alzheimer and other dementias
SCD: subjective cognitive decline
STARD: Standards for Reporting of Diagnostic Accuracy Studies
t-tau: total tau
WER: word error rate
