# Supplementary material for: CogniPaz, an Ecologically Oriented Gamified mHealth Tool for Detecting Mild Cognitive Impairment: Prospective Cohort Diagnostic Accuracy Study
Source: JMIR Mhealth Uhealth. 2026 Nov 4;14:e99688. doi: 10.2196/99688 (PMC13734706; doi:10.2196/99688)
Supplement: Supplementary file 1 [file mhealth-v14-e99688-s006.pdf]

STARD 2015 Checklist

No	Item	Reported on page #
**TITLE OR ABSTRACT**		
1	Identification as a study of diagnostic accuracy using at least one measure of accuracy (such as sensitivity, specificity, predictive values, or AUC)	1–2
**ABSTRACT**		
2	Structured summary of study design, methods, results, and conclusions (for specific guidance, see STARD for Abstracts)	1–2
**INTRODUCTION**		
3	Scientific and clinical background, including the intended use and clinical role of the index test	2
4	Study objectives and hypotheses	1–2
**METHODS**		
5	Study design — Whether data collection was planned before the index test and reference standard were performed (prospective study) or after (retrospective study)	1, 4
6	Participants — Eligibility criteria	4
7	Participants — On what basis potentially eligible participants were identified (such as symptoms, results from previous tests, inclusion in registry)	4–5
8	Participants — Where and when potentially eligible participants were identified (setting, location and dates)	1, 4–5
9	Participants — Whether participants formed a consecutive, random or convenience series	5
10a	Test methods — Index test, in sufficient detail to allow replication	3–5
10b	Test methods — Reference standard, in sufficient detail to allow replication	4–5, 7, 10
11	Test methods — Rationale for choosing the reference standard (if alternatives exist)	4–5
12a	Test methods — Definition of and rationale for test positivity cut-offs or result categories of the index test, distinguishing pre-specified from exploratory	6, 11, 13–15, 19
12b	Test methods — Definition of and rationale for test positivity cut-offs or result categories of the reference standard, distinguishing pre-specified from exploratory	5
13a	Test methods — Whether clinical information and reference standard results were available to the performers/readers of the index test	5
13b	Test methods — Whether clinical information and index test results were available to the assessors of the reference standard	5
14	Analysis — Methods for estimating or comparing measures of diagnostic accuracy	6
15	Analysis — How indeterminate index test or reference standard results were handled	9
16	Analysis — How missing data on the index test and reference standard were handled	5–7, 15
17	Analysis — Any analyses of variability in diagnostic accuracy, distinguishing pre-specified from exploratory	6, 10–13, 15–16
18	Analysis — Intended sample size and how it was determined	5

No	Item	Reported on page #
**RESULTS**		
19	Participants — Flow of participants, using a diagram	7
20	Participants — Baseline demographic and clinical characteristics of participants	8
21a	Participants — Distribution of severity of disease in those with the target condition	5, 8
21b	Participants — Distribution of alternative diagnoses in those without the target condition	Not applicable to this design
22	Participants — Time interval and any clinical interventions between index test and reference standard	5
23	Test results — Cross tabulation of the index test results (or their distribution) by the results of the reference standard	15
24	Test results — Estimates of diagnostic accuracy and their precision (such as 95% confidence intervals)	10–13, 15
25	Test results — Any adverse events from performing the index test or the reference standard	16
**DISCUSSION**		
26	Study limitations, including sources of potential bias, statistical uncertainty, and generalisability	18–19
27	Implications for practice, including the intended use and clinical role of the index test	17–18, 20
**OTHER INFORMATION**		
28	Registration number and name of registry	Observational study, not registered.
29	Where the full study protocol can be accessed	Not reported
30	Sources of funding and other support; role of funders	20
