# Supplementary material for: CogniPaz, an Ecologically Oriented Gamified mHealth Tool for Detecting Mild Cognitive Impairment: Prospective Cohort Diagnostic Accuracy Study
Source: JMIR Mhealth Uhealth. 2026 Nov 4;14:e99688. doi: 10.2196/99688 (PMC13734706; doi:10.2196/99688)
Supplement: Supplementary file 2 [file mhealth-v14-e99688-s003.zip › COGNIPAZ_sanitized/src/app/home/doctor-questionnaire/doctor-questionnaire.page.html]

# **Algoritmo de toma de decisiones**

# ¿El principal motivo de consulta del paciente son los síntomas cognitivos?

  


SÍ


NO
  
  

### Valorar los síntomas principales del paciente antes de plantear la derivación. El paciente no es candidato a participar el proceso de validación de la aplicación


# **Algoritmo de toma de decisiones**

# ¿Los síntomas cognitivos del paciente producen dependencia para sus actividades cotidianas o está ya diagnosticado de deterioro cognitivo y/o demencia?

  


SÍ


NO
  

### El paciente tiene ya una demencia clínicamente establecida, por lo que no sería candidato a esta vía de derivación. En caso de tener criterios de demencia moderada, > 70 años y pluripatología, valorar remitir a Geriatría


# **Algoritmo de toma de decisiones - Checklist**

# ¿Entre los antecedentes personales del paciente, existe alguna causa que potencialmente pueda explicar los síntomas cognitivos del mismo?


  
  


### - Daño cerebral previo (traumatismo, ictus)


SÍ


NO


### - Enfermedades neurológicas previas que afecten al sistema nervioso central (epilepsia, esclerosis múltiple, tumores, …)


SÍ


NO


### - Enfermedad psiquiátrica activa y en tratamiento (depresión mayor, trastorno bipolar, ..)


SÍ


NO


### - Consumo excesivo de alcohol, cocaína u otras drogas de abuso (excluyendo tabaco)


SÍ


NO


### - Consumo diario de alguno de los siguientes grupos farmacológicos: opiáceos, benzodiacepinas, antidepresivos tricíclicos o antiepilépticos.


SÍ


NO
  
  


# Si existe sospecha de Deterioro Cognitivo Leve, realizar e-consulta.


# Administre el test al paciente a través de la tablet. Asegúrese de que el paciente tiene gafas y audífonos colocados en caso de que los necesite. Algunos pacientes podrían necesitar ayuda para el manejo de la herramienta

  
ADMINISTRAR TEST
