# Supplementary material for: CogniPaz, an Ecologically Oriented Gamified mHealth Tool for Detecting Mild Cognitive Impairment: Prospective Cohort Diagnostic Accuracy Study
Source: JMIR Mhealth Uhealth. 2026 Nov 4;14:e99688. doi: 10.2196/99688 (PMC13734706; doi:10.2196/99688)
Supplement: Supplementary file 2 [file mhealth-v14-e99688-s003.zip › COGNIPAZ_sanitized/src/app/home/test/end/end.page.html]

**¡¡Enhorabuena!!**

- # Ha completado la aplicación
- # La puntuación obtenida no es indicativa de ningún diagnóstico y tan sólo dará a su médico de atención primaria las indicaciones oportunas para derivar su caso a consultas de Neurología
- # Ya puede devolver la tableta a su Médico de Atención Primaria.

### **Hacer encuesta de usabilidad**


- ### **Percepción general de la aplicación (1=Aburrida; 9=Interesante)**

  **{{ perception }}**
  
- ### **Dificultad de uso de la aplicación (1=Difícil de usar; 9=Fácil de usar)**

  **{{ difficulty }}**
  
- ### **La información mostrada en la pantalla parece (1=Ilógica/Poco entendible; 9=Lógica/Entendible)**

  **{{ logical }}**
  
- ### **Las tareas que se realizan en la aplicación resultan (1=Confusas; 9=Claras)**

  **{{ tasks\_information }}**
  
- ### **El tiempo que conlleva realizar las tareas resulta (1=Largo; 9=Corto y adecuado)**

  **{{ tasks\_duration }}**
  
- ### **Las tareas resultan (1=Muy difíciles; 9=Muy sencillas)**

  **{{ tasks\_difficulty }}**
  
- ### **Percepción de utilidad de la aplicación (1=Poco útil; 9=Muy útil)**

  **{{ usability }}**
  
- Otros comentarios:


Siguiente


# Debe rellenar los siguientes datos:

  
  

## **FORMULARIO COMÚN PARA TODOS LOS PARTICIPANTES**

- Tipo de usuario

  Paciente
  Control
- Ámbito de realización

  Atención primaria
  Consulta neurología general
  Consulta de trastornos cognitivos
  Otros
- Sexo

  Varón
  Mujer
- Nivel educativo

  Analfabeto
  Estudios primarios incompletos
  Estudios primarios
  Estudios secundarios
  Bachillerato
  Estudios universitarios incompletos
  Estudios universitarios
  Formación profesional
- Sí
  No
- MoCa

  MMSE

  - ### Funciones ejecutivas

    **{{ funciones\_ejecutivas }}**
  - ### Denominación

    **{{ denominacion }}**
  - ### Atención

    **{{ atencion }}**
  - ### Lenguaje

    **{{ lenguaje }}**
  - ### Abstracción

    **{{ abstraccion }}**
  - ### Memoria

    **{{ memoria }}**
  - ### Orientación

    **{{ orientacion }}**
    

  # TOTAL: {{sum\_MoCa}}/30

  - ### Orientación temporal

    **{{ orientacion\_temporal }}**
  - ### Orientación espacial

    **{{ orientacion\_espacial }}**
  - ### Fijación

    **{{ fijacion }}**
  - ### Atención

    **{{ atencion\_MMSE }}**
  - ### Memoria

    **{{ memoria\_MMSE }}**
  - ### Lenguaje

    **{{ lenguaje\_MMSE }}**
  - ### Orden

    **{{ orden }}**
  - ### Escritura

    **{{ escritura }}**
  - ### Lectura

    **{{ lectura }}**
  - ### Dibujo

    **{{ dibujo }}**
    

  # TOTAL: {{sum\_MMSE}}/30
- Sí
  No
- Tarea 1 - Lista de la compra
  Tarea 2 - Recorrido en metro
  Tarea 3.1 - Noticia de televisión
  Tarea 3 - Calendario
  Tarea 4 - Números directos
  Tarea 5 - Números inversos
  Tarea 6 - Secuencia
  Tarea 7 - Supermercado
  Tarea 8 - Cálculo
  Tarea 9 - Noticia
- Observaciones:

## **FORMULARIO PARA PACIENTES**

- Memoria
  Orientación
  Atención
  Lenguaje
  Habilidades visuoespaciales
  Funciones ejecutivas
- Quejas cognitivas subjetivas
  Deterioro cognitivo leve
- Sí
  No
- Pruebas complementarias realizadas

    
  - Resonancia magnética
  - Infartos lacunares
  - Infartos territoriales
  - Atrofia hipocampal
  - Normal
  - PET-FDG cerebral

  Regiones con hipometabolismo

  - Frontal
  - Parietal
  - Temporal
  - Occipital
  - Normal
  - Biomarcadores en LCR
  - Compatibles con E. Alzheimer
  - PET de amiloide
  - Positivo
  - Negativo


Siguiente


**RESULTADOS**

## ID DEL PACIENTE: {{id}}

## PUNTUACIONES: ({{score1Tarea1}}-{{score2Tarea1}})-{{scoreTarea2}}-{{scoreTarea31}}-{{scoreTarea3}}-{{scoreTarea4}}-{{scoreTarea5}}-{{scoreTarea6}}-({{score1Tarea7}}-{{score2Tarea7}})-{{scoreTarea8}}-{{scoreTarea9}}

- ## **Todo paciente que haya completado la aplicación será remitido a Neurología a través de e-consulta indicando como motivo de consulta síntomas cognitivos y copiando las puntuaciones que aparecen en la pantalla.**
- ## En función de estas puntuaciones, desde Neurología se decidirá si la cita se gestiona a través del circuito habitual (Neurología general) o mediante la vía directa a la Unidad de Trastornos Cognitivos.
- ## Muchas gracias por su participación.

SALIR
