# Supplementary material for: CogniPaz, an Ecologically Oriented Gamified mHealth Tool for Detecting Mild Cognitive Impairment: Prospective Cohort Diagnostic Accuracy Study
Source: JMIR Mhealth Uhealth. 2026 Nov 4;14:e99688. doi: 10.2196/99688 (PMC13734706; doi:10.2196/99688)
Supplement: Supplementary file 2 [file mhealth-v14-e99688-s003.zip › COGNIPAZ_sanitized/src/app/home/test/tarea1/tarea1.page.html]

**TAREA 1 - Lista de la compra**

# Imagínese que hoy tiene que hacer la compra en el supermercado. Al pulsar en siguiente, en la pantalla van a aparecer los elementos de la lista de la compra que usted tendrá que memorizar. Mantenga la atención y, al final, escríbalos en la pantalla.

# Pulse en siguiente cuando esté preparado.

Siguiente


# {{currentWord}}


# Escriba todos los elementos de la lista de la compra que recuerde. No se preocupe por los acentos o las faltas de ortografía. Cuando acabe de escribir la palabra, pulse el botón "AÑADIR "

  

# Escribe todas las palabras de las que te acuerdes:

  


AÑADIR 

# Palabra ya incluida!


# Palabras registradas:

- ### {{ word }}


  Eliminar

CONTINUAR


# A continuación, van a aparecer de nuevo los elementos de la lista de la compra. Intente memorizarlos y escribirlos al final. Recuerde la lista de la compra porque al final del juego tendrá que comprar en el supermercado

  

# Pulse siguiente cuando esté preparado

Siguiente
