# Supplementary material for: CogniPaz, an Ecologically Oriented Gamified mHealth Tool for Detecting Mild Cognitive Impairment: Prospective Cohort Diagnostic Accuracy Study
Source: JMIR Mhealth Uhealth. 2026 Nov 4;14:e99688. doi: 10.2196/99688 (PMC13734706; doi:10.2196/99688)
Supplement: Supplementary file 2 [file mhealth-v14-e99688-s003.zip › COGNIPAZ_sanitized/src/app/home/test/tarea1-low/tarea1-low.page.html]

**TAREA 1 - Lista de la compra**

# Imagínese que hoy tiene que hacer la compra en el supermercado. Al pulsar en siguiente, en la pantalla van a aparecer los elementos de la lista de la compra que usted tendrá que memorizar. Mantenga la atención y, al final, selecciónelos en la pantalla.

# Pulse en siguiente cuando esté preparado.

Siguiente


# Pulse sobre los elementos de la compra que habían aparecido anteriormente. Cuando haya terminado, pulse en "CONTINUAR".

CONTINUAR


# A continuación, van a aparecer de nuevo los elementos de la lista de la compra. Intente memorizarlos y seleccionarlos al final. Recuerde la lista de la compra porque al final del juego tendrá que comprar en el supermercado.

  

# Pulse en Siguiente cuando esté preparado.

Siguiente
