# Supplementary material for: CogniPaz, an Ecologically Oriented Gamified mHealth Tool for Detecting Mild Cognitive Impairment: Prospective Cohort Diagnostic Accuracy Study
Source: JMIR Mhealth Uhealth. 2026 Nov 4;14:e99688. doi: 10.2196/99688 (PMC13734706; doi:10.2196/99688)
Supplement: Supplementary file 2 [file mhealth-v14-e99688-s003.zip › COGNIPAZ_sanitized/src/app/home/test/tarea1-voz/tarea1-voz.page.html]

**TAREA 1 - Lista de la compra**

# Imagínese que hoy tiene que hacer la compra en el supermercado. Al pulsar en siguiente, en la pantalla van a aparecer los elementos de la lista de la compra que usted tendrá que memorizar.

# Pulse en siguiente cuando esté preparado.

Siguiente


# {{currentWord}}


# Diga todos los elementos de la lista de la compra que recuerde. Debe pulsar el botón "AÑADIR " y decir en voz alta los elementos cuando vea el dibujo del micrófono. Estos aparecerán escritos en la pantalla. Repita este proceso si es necesario para decir todos los elementos que recuerde.

  

AÑADIR 

# Palabra ya incluida!


# Palabras registradas:

- ### {{ word }}


  Eliminar

CONTINUAR


# A continuación, van a aparecer de nuevo los elementos de la lista de la compra. Intente memorizarlos y escribirlos al final. Recuerde la lista de la compra porque al final del juego tendrá que comprar en el supermercado

  

# Pulse siguiente cuando esté preparado

Siguiente
