# Supplementary material for: CogniPaz, an Ecologically Oriented Gamified mHealth Tool for Detecting Mild Cognitive Impairment: Prospective Cohort Diagnostic Accuracy Study
Source: JMIR Mhealth Uhealth. 2026 Nov 4;14:e99688. doi: 10.2196/99688 (PMC13734706; doi:10.2196/99688)
Supplement: Supplementary file 2 [file mhealth-v14-e99688-s003.zip › COGNIPAZ_sanitized/src/app/home/test/tarea2/tarea2.page.html]

**TAREA 2 - Recorrido en metro**

# Antes de hacer la compra en el supermercado, nos han surgido algunas tareas que tenemos que llevar a cabo. Para ello, vamos a tener que utilizar el metro para desplazarnos.

# Pulse en siguiente cuando esté preparado.

Siguiente


# Si usted vive en Cuatro Caminos, señale las estaciones de metro por las que tendría que pasar para llegar de la forma con menor número de estaciones intermedias al Hospital La Paz (estación de metro Begoña).

  

# Para señalar una estación, pulse encima de ella en el plano de metro.

  

# Pulse en siguiente cuando haya terminado.


Siguiente
