# Supplementary material for: CogniPaz, an Ecologically Oriented Gamified mHealth Tool for Detecting Mild Cognitive Impairment: Prospective Cohort Diagnostic Accuracy Study
Source: JMIR Mhealth Uhealth. 2026 Nov 4;14:e99688. doi: 10.2196/99688 (PMC13734706; doi:10.2196/99688)
Supplement: Supplementary file 2 [file mhealth-v14-e99688-s003.zip › COGNIPAZ_sanitized/src/app/home/test/tarea3/tarea3.page.html]

**TAREA 3 - Calendario**

# Seleccione el día, mes y año en el que estamos.

- ### **Día**

  {{ d }}
- ### **Mes**

  {{ m.name }}
- ### **Año**

  {{ y }}


Siguiente
