# Supplementary material for: CogniPaz, an Ecologically Oriented Gamified mHealth Tool for Detecting Mild Cognitive Impairment: Prospective Cohort Diagnostic Accuracy Study
Source: JMIR Mhealth Uhealth. 2026 Nov 4;14:e99688. doi: 10.2196/99688 (PMC13734706; doi:10.2196/99688)
Supplement: Supplementary file 2 [file mhealth-v14-e99688-s003.zip › COGNIPAZ_sanitized/src/app/home/test/tarea3-1/tarea3-1/tarea3-1.page.html]

**TAREA 3.1 - Noticia de televisión**

# Mientras espera el metro, en la televisión aparece una noticia que le llama la atención y que quiere recordar para decírsela posteriormente a su hijo.

# Preste atención a la noticia porque le haremos preguntas sobre ella. Cuando esté preparado, pulse sobre la televisión.

# Cuando esté preparado, pulse sobre la televisión.


# Recuerda la noticia que escuchó mientras esperaba al metro… Intente contarle a su hijo todos los detalles que recuerde.


- #### **¿Cuántos rescates hubo?**
- #### **¿A qué distancia en metros estaba el parque de bomberos?**
- ### **¿A qué hora de la noche sucedió?**

  {{ h }}


Siguiente
