# Supplementary material for: CogniPaz, an Ecologically Oriented Gamified mHealth Tool for Detecting Mild Cognitive Impairment: Prospective Cohort Diagnostic Accuracy Study
Source: JMIR Mhealth Uhealth. 2026 Nov 4;14:e99688. doi: 10.2196/99688 (PMC13734706; doi:10.2196/99688)
Supplement: Supplementary file 2 [file mhealth-v14-e99688-s003.zip › COGNIPAZ_sanitized/src/app/home/test/tarea4/tarea4.page.html]

**TAREA 4 - Números directos**

# En la pantalla van a aparecer una serie de números. Fíjese bien porque luego tendrá que escribirlos en el mismo orden.

# Pulse en Siguiente cuando esté preparado.


Siguiente


# {{currentNumber}}


# Escriba seguidos los números que ha visto en el orden en el que han aparecido. Cuando haya acabado, pulse en Continuar.

  

CONTINUAR
