# Supplementary material for: CogniPaz, an Ecologically Oriented Gamified mHealth Tool for Detecting Mild Cognitive Impairment: Prospective Cohort Diagnostic Accuracy Study
Source: JMIR Mhealth Uhealth. 2026 Nov 4;14:e99688. doi: 10.2196/99688 (PMC13734706; doi:10.2196/99688)
Supplement: Supplementary file 2 [file mhealth-v14-e99688-s003.zip › COGNIPAZ_sanitized/src/app/home/test/tarea6/tarea6.page.html]

**TAREA 6 - Secuencia**

# En la pantalla va a ver 9 bolas de color verde. Las bolas van a cambiar de color siguiendo una secuencia que usted tendrá que recordar al final.

# Pulse en siguiente cuando esté preparado.

Siguiente


# Señale la secuencia pulsando en cada botón para que cambie de color.
