# Supplementary material for: CogniPaz, an Ecologically Oriented Gamified mHealth Tool for Detecting Mild Cognitive Impairment: Prospective Cohort Diagnostic Accuracy Study
Source: JMIR Mhealth Uhealth. 2026 Nov 4;14:e99688. doi: 10.2196/99688 (PMC13734706; doi:10.2196/99688)
Supplement: Supplementary file 2 [file mhealth-v14-e99688-s003.zip › COGNIPAZ_sanitized/src/app/home/test/tarea7/tarea7.page.html]

**TAREA 7 - Supermercado**

# Ha llegado al supermercado y tiene que recordar la lista de la compra que aprendió al principio.

Siguiente


## Diga aquellos elementos que recuerde. Para decirlos, pulse en "AÑADIR". Pulse en siguiente cuando haya terminado.

AÑADIR


# Palabra ya incluida!

# Palabras registradas:

- ### {{ word }}


  Eliminar


Siguiente


### Aquí puede ver el plano del supermercado. Señale las secciones donde están los productos que tiene que comprar y elija el adecuado. Cuando termine, pulse en la caja:

{{shape.name}}

## {{selectedShape.name}}

Volver

# **CAJA**


X
