# Supplementary material for: CogniPaz, an Ecologically Oriented Gamified mHealth Tool for Detecting Mild Cognitive Impairment: Prospective Cohort Diagnostic Accuracy Study
Source: JMIR Mhealth Uhealth. 2026 Nov 4;14:e99688. doi: 10.2196/99688 (PMC13734706; doi:10.2196/99688)
Supplement: Supplementary file 2 [file mhealth-v14-e99688-s003.zip › COGNIPAZ_sanitized/src/app/home/test/test.page.html]

# **Por favor, antes de comenzar asegúrese de de que la Tablet está conectada a Internet**

Ver datos offline

{{error}}

## Datos Offline en JSON:

```
{{ jsonData }}
```

Borrar datos offline

Siguiente


# **COGNIPAZ**

- # La herramienta COGNIPAZ ha sido desarrollada para facilitar la derivación de pacientes con síntomas cognitivos desde atención primaria a las consultas específicas de trastornos cognitivos en el servicio de Neurología.
  
- # **No se trata de una herramienta diagnóstica y, por lo tanto, no se preocupe si comete fallos.**

  

  


Activar audioguía

Siguiente


# Le vamos a pedir diez minutos de atención en una serie de tareas que realizará a través de la Tablet.

# Cada tarea tiene un tiempo asignado, por lo que usted no tendrá que pulsar ningún botón para pasar de una tarea a otra salvo que se le indique.

# Cuando esté preparado para empezar, pulse el botón “Siguiente”

Siguiente
