# Supplementary material for: CogniPaz, an Ecologically Oriented Gamified mHealth Tool for Detecting Mild Cognitive Impairment: Prospective Cohort Diagnostic Accuracy Study
Source: JMIR Mhealth Uhealth. 2026 Nov 4;14:e99688. doi: 10.2196/99688 (PMC13734706; doi:10.2196/99688)
Supplement: Supplementary file 3 [file mhealth-v14-e99688-s004.docx]

## Supplementary Table S1. Threshold-performance table across candidate cutoffs (24.5, 26.5, 28.5) for all clinical and biomarker comparisons, with 95% confidence intervals.

| Comparison | Cutoff | TP | TN | FP | FN | Sens, % (95% CI) | Spec, % (95% CI) | PPV, % (95% CI) | NPV, % (95% CI) | LR+ (95% CI) | LR- (95% CI) | Acc, % (95% CI) |
| --- | --- | --- | --- | --- | --- | --- | --- | --- | --- | --- | --- | --- |
| MCI vs HC | 24.5 | 26 | 25 | 2 | 14 | 65.0 (48.3-79.4) | 92.6 (75.7-99.1) | 92.9 (76.5-99.1) | 64.1 (47.2-78.8) | 8.78 (2.27-33.95) | 0.38 (0.24-0.58) | 76.1 (64.1-85.7) |
| MCI vs HC | 26.5 | 30 | 24 | 3 | 10 | 75.0 (58.8-87.3) | 88.9 (70.8-97.6) | 90.9 (75.7-98.1) | 70.6 (52.5-84.9) | 6.75 (2.29-19.91) | 0.28 (0.16-0.49) | 80.6 (69.1-89.2) |
| MCI vs HC | 28.5 | 34 | 20 | 7 | 6 | 85.0 (70.2-94.3) | 74.1 (53.7-88.9) | 82.9 (67.9-92.8) | 76.9 (56.4-91.0) | 3.28 (1.71-6.28) | 0.20 (0.09-0.44) | 80.6 (69.1-89.2) |
| aMCI vs HC | 24.5 | 17 | 25 | 2 | 5 | 77.3 (54.6-92.2) | 92.6 (75.7-99.1) | 89.5 (66.9-98.7) | 83.3 (65.3-94.4) | 10.43 (2.70-40.35) | 0.25 (0.11-0.53) | 85.7 (72.8-94.1) |
| aMCI vs HC | 26.5 | 18 | 24 | 3 | 4 | 81.8 (59.7-94.8) | 88.9 (70.8-97.6) | 85.7 (63.7-97.0) | 85.7 (67.3-96.0) | 7.36 (2.49-21.79) | 0.20 (0.08-0.50) | 85.7 (72.8-94.1) |
| aMCI vs HC | 28.5 | 20 | 20 | 7 | 2 | 90.9 (70.8-98.9) | 74.1 (53.7-88.9) | 74.1 (53.7-88.9) | 90.9 (70.8-98.9) | 3.51 (1.83-6.72) | 0.12 (0.03-0.47) | 81.6 (68.0-91.2) |
| MCI vs SCD | 24.5 | 26 | 16 | 1 | 14 | 65.0 (48.3-79.4) | 94.1 (71.3-99.9) | 96.3 (81.0-99.9) | 53.3 (34.3-71.7) | 11.05 (1.63-75.0) | 0.37 (0.24-0.58) | 73.7 (60.3-84.5) |
| MCI vs SCD | 26.5 | 30 | 14 | 3 | 10 | 75.0 (58.8-87.3) | 82.4 (56.6-96.2) | 90.9 (75.7-98.1) | 58.3 (36.6-77.9) | 4.25 (1.50-12.05) | 0.30 (0.17-0.54) | 77.2 (64.2-87.3) |
| MCI vs SCD | 28.5 | 34 | 14 | 3 | 6 | 85.0 (70.2-94.3) | 82.4 (56.6-96.2) | 91.9 (78.1-98.3) | 70.0 (45.7-88.1) | 4.82 (1.71-13.56) | 0.18 (0.08-0.39) | 84.2 (72.1-92.5) |
| aMCI vs SCD | 24.5 | 17 | 16 | 1 | 5 | 77.3 (54.6-92.2) | 94.1 (71.3-99.9) | 94.4 (72.7-99.9) | 76.2 (52.8-91.8) | 13.14 (1.94-89.15) | 0.24 (0.11-0.53) | 84.6 (69.5-94.1) |
| aMCI vs SCD | 26.5 | 18 | 14 | 3 | 4 | 81.8 (59.7-94.8) | 82.4 (56.6-96.2) | 85.7 (63.7-97.0) | 77.8 (52.4-93.6) | 4.64 (1.63-13.19) | 0.22 (0.09-0.55) | 82.1 (66.5-92.5) |
| aMCI vs SCD | 28.5 | 20 | 14 | 3 | 2 | 90.9 (70.8-98.9) | 82.4 (56.6-96.2) | 87.0 (66.4-97.2) | 87.5 (61.7-98.4) | 5.15 (1.83-14.51) | 0.11 (0.03-0.42) | 87.2 (72.6-95.7) |
| Confirmed ND vs Unconfirmed ND | 24.5 | 12 | 18 | 6 | 3 | 80.0 (51.9-95.7) | 75.0 (53.3-90.2) | 66.7 (41.0-86.7) | 85.7 (63.7-97.0) | 3.20 (1.53-6.69) | 0.27 (0.09-0.75) | 76.9 (60.7-88.9) |
| Confirmed ND vs Unconfirmed ND | 26.5 | 13 | 13 | 11 | 2 | 86.7 (59.5-98.3) | 54.2 (32.8-74.4) | 54.2 (32.8-74.4) | 86.7 (59.5-98.3) | 1.89 (1.17-3.05) | 0.25 (0.06-0.94) | 66.7 (49.8-80.9) |
| Confirmed ND vs Unconfirmed ND | 28.5 | 13 | 10 | 14 | 2 | 86.7 (59.5-98.3) | 41.7 (22.1-63.4) | 48.1 (28.7-68.1) | 83.3 (51.6-97.9) | 1.49 (1.00-2.20) | 0.32 (0.08-1.26) | 59.0 (42.1-74.4) |

CI: confidence interval, TP: true positives; TN: true negatives; FP: false positives; FN: false negatives; PPV: positive predictive value; NPV: negative predictive value; LR⁺: positive likelihood ratio; LR⁻: negative likelihood ratio; MCI: mild cognitive impairment; HC: healthy controls; aMCI: amnestic mild cognitive impairment; ND: neurodegenerative; SCD: subjective cognitive decline; Sens: sensitivity; Spec: specificity.

Sensitivity, specificity, PPV, NPV, and accuracy: 95% CI by Clopper-Pearson exact method. LR+/LR-: 95% CI by the log-normal method.
