# Supplementary material for: CogniPaz, an Ecologically Oriented Gamified mHealth Tool for Detecting Mild Cognitive Impairment: Prospective Cohort Diagnostic Accuracy Study
Source: JMIR Mhealth Uhealth. 2026 Nov 4;14:e99688. doi: 10.2196/99688 (PMC13734706; doi:10.2196/99688)
Supplement: Supplementary file 4 [file mhealth-v14-e99688-s002.DOC]

**Multimedia Appendix 2. Technical software documentation for the CogniPaz application**

# 1. Purpose of this technical appendix

This appendix documents the software implementation of the CogniPaz application, including hardware and operating environment, software stack, architecture, navigation and task lifecycle, speech-recognition processing, scoring model, application data model, persistence and synchronization logic, backend interaction and Android build pipeline. The document is purely technical and focused on the application itself.

# 2. Hardware and operating environment

| Component | Specification |
| --- | --- |
| Study device | Lenovo Tab M9 |
| Screen | 9-inch touchscreen |
| Processor | MediaTek Helio G80 |
| Memory | 3 GB RAM |
| Storage | 32 GB expandable internal storage |
| Connectivity | Wi-Fi; Bluetooth 5.1 |
| Audio input/output | Integrated microphone and integrated speaker |
| Operating system during study | Android 12 |
| Deployment mode | Minimally supervised clinical administration |
| Intended study setting | Quiet room at the Cognitive Disorders Unit |
| Remote/unsupervised use | Not evaluated in this study |

# 3. Software Architecture and Data Flow Framework

CogniPaz utilizes a robust tablet front-end coupled with an institutional backend architecture. As detailed in the software environment summary table below, the application is built from an Ionic/Angular source project, packaged through Capacitor into an Android project, and compiled using the Android Gradle Plugin. The native tablet application governs the patient-facing user interface, task-presentation logic, independent task timers, and local session variables.

| Layer | Technology/version |
| --- | --- |
| Application type | Android tablet app |
| Front-end framework | Angular 18.2.5 |
| Mobile UI framework | Ionic Angular 8.3.1 |
| Native bridge/runtime | Capacitor Core/Android/CLI 6.1.2 |
| Speech-recognition plugin | Cordova SpeechRecognition plugin 1.2.0 |
| Ionic Native wrapper | Ionic Native Speech Recognition 5.36.0 |
| Local storage | Ionic Storage Angular 4.0.0 |
| Reactive library | RxJS 7.8.1 |
| TypeScript | 5.4.5 |
| Android Gradle Plugin | 8.2.1 |
| Android min SDK | 22 |
| Android compile SDK | 34 |
| Android target SDK | 34 |
| Application ID | org.research.cognipaz |

At session initiation, the application generates a unique internal study record identifier, which serves as the app-level key for tracking metrics. Upon the conclusion of each cognitive task, the application packages the task-specific score, error counts, completion time, and session identifier into a structured data object. These data are progressively transmitted to the institutional backend via secure HTTPS POST requests directed to a REDCap-compatible API using record-level JSON/flat-format parameters. To prevent data loss from temporary connectivity drops in clinical environments, an automated offline contingency mechanism evaluates network status prior to any API transaction. If an active connection is available, data are transmitted immediately. If connectivity is disrupted, the data object is queued locally within an encrypted temporary partition utilizing Ionic Storage; upon network restoration, a sync service flushes this queue to the backend, removing local objects only after receiving a successful transmission confirmation. Crucially, the platform is strictly bounded to store and transmit only processed variables (task metrics, usability responses, and clinician-entered data), adhering to strict data minimization standards.

The speech recognition module is utilized exclusively during verbal recall tasks and is implemented via the Android speech recognition API through a Cordova/Ionic plugin configured for Spanish (es-ES). Upon activation from the task interface, the application processes the textual hypothesis returned by Android by performing uppercase conversion and accent normalization. String matching against the target list is then executed using a Levenshtein distance threshold of $\leq$1.

To ensure compliance with participant privacy standards, the architecture guarantees that no audio recordings, raw text strings, or intermediate correction logs are written to local storage or transmitted to the backend. Because no parallel manual item-level scoring log or raw audio files were retained, exact automated-versus-manual scoring agreement (e.g., Word Error Rate) cannot be retrospectively calculated. To mitigate automated errors in real time during the study, several live user-interface safeguards were implemented in the UI workflow: (1) speech recognition was used strictly as a transient input; (2) successfully recognized words were immediately rendered on-screen for verification prior to final submission; (3) incorrect words could be manually deleted by the participant before confirming the task; (4) speech-recognition attempts could be repeated under minimal supervisor oversight; and (5) near-duplicate words were algorithmically ignored. All examiner assistance provided during this process was subsequently recorded via an in-app check.

# 4. Application documentation diagrams

The following figures provide the principal architecture, interaction and process views required to document the application. The title embedded in each diagram matches the figure caption and the ordering in this document.

## Figure A1. Runtime context and deployment architecture.

**Figure A1. Runtime context and deployment architecture.** This diagram shows the runtime environment of the application during supervised clinical use, including the participant, clinician/researcher, Android tablet, institutional backend and analytical output.

## Figure A2. Black-box application architecture.

**Figure A2. Black-box application architecture.** This black-box view represents the main external actors and interfaces of CogniPaz: participant interaction through the tablet, clinician oversight, data transmission to the institutional backend and export to the analytical dataset.

## Figure A3. White-box component architecture.

**Figure A3. White-box component architecture.** This white-box view describes the main internal software modules of the tablet application, including the Ionic/Angular user interface, routing, task screens, scoring logic, timers, speech-recognition module, local storage and backend communication services.

## Figure A4. Clinical use-case diagram.

**Figure A4. Clinical use-case diagram.** This use-case diagram summarizes the principal interactions between participant, examiner and institutional backend during a CogniPaz assessment session.

## Figure A5. Assessment navigation workflow.

**Figure A5. Assessment navigation workflow.** This flow diagram represents the navigation logic of the application across the task sequence from session start to final score display.

## Figure A6. Standard task lifecycle.

**Figure A6. Standard task lifecycle.** This diagram shows the lifecycle of an individual task, including initialization, instruction display, user interaction, response validation, score calculation, time recording and persistence.

## Figure A7. Speech-recognition module workflow.

**Figure A7. Speech-recognition module workflow.** This flow diagram describes the internal processing pipeline for speech-based tasks, from user initiation to text recognition, normalization, matching, scoring and UI confirmation.

## Figure A8. Scoring model and total-score aggregation.

**Figure A8. Scoring model and total-score aggregation.** This diagram shows the relation between raw user responses, task-specific scoring logic, error accounting, timing capture and total-score aggregation.

## Figure A9. Application data model and variable domains.

**Figure A9. Application data model and variable domains.** This diagram summarizes the principal domains of structured data handled by the application, including record/session variables, task scores, errors, times, usability fields and clinical metadata.

## Figure A10. Persistence and synchronization workflow.

**Figure A10. Persistence and synchronization workflow.** This flow diagram represents the persistence model of the application, including immediate HTTPS transmission, offline temporary storage and later synchronization.

## Figure A11. Backend transaction sequence.

**Figure A11. Backend transaction sequence.** This sequence diagram illustrates the order of interactions between the application and the backend for record creation, task-level updates and final session data submission.

## Figure A12. Build and Android packaging pipeline.

**Figure A12. Build and Android packaging pipeline.** This diagram summarizes the build pipeline from source code and front-end dependencies to the Capacitor/Android project and packaged application.

# 5. Task-level scoring summary

| Task | Main construct | Input mode | Deterministic scoring rule | Score range | Stored error variables |
| --- | --- | --- | --- | --- | --- |
| Task 1 round 1 | Immediate verbal learning | Speech recognition | 1 point per unique target word recalled (plátanos, azúcar, leche, hamburguesas, salmón, lejía). Normalized via Levenshtein distance ≤1. | 0-6 | Count of unique non-target words. |
| Task 1 round 2 | Repeated verbal learning | Speech recognition | Second exposure to the same 6-word list. Coded and scored independently using identical Levenshtein parameters. | 0-6 | Count of unique non-target words. |
| Task 2 | Planning & visuospatial navigation | Touchscreen | 1 point if the shortest metro path is selected exactly (Nuevos Ministerios, Santiago Bernabéu, Cuzco, Plaza de Castilla, Chamartín, Begoña). 0 points otherwise. | 0-1 | Count of unnecessary selected stations. |
| Task 3.1 | Immediate contextual recall | Numeric input | 1 point per correct detail provided from the broadcast: 12 rescues, 400 meters, and 11 p.m. / 23:00. | 0-3 | Not stored. |
| Task 3.2 | Temporal orientation | Touchscreen | 1 point if the entered day, month, and year exactly match the current device date on the assessment day. | 0-1 | Not stored. |
| Task 4 | Forward digit span | Numeric input | 1 point if the 5-digit sequence is entered in identical sequential order (58472). Dichotomous pass/fail. | 0-1 | Not stored. |
| Task 5 | Backward digit span | Numeric input | 1 point if the 3-digit sequence is entered in exact reverse order (193). Dichotomous pass/fail. | 0-1 | Not stored. |
| Task 6 | Visuospatial sequencing | Touchscreen | 1 point if the 5-position sequence shown on the 3x3 array is reproduced in exact chronological order. | 0-1 | Not stored. |
| Task 7 free recall | Delayed verbal recall | Speech recognition | Delayed retrieval of the original 6-word shopping list. 1 point per unique target word verified using Levenshtein distance ≤1. 1-point penalty per incorrect response. | 0-6 | Count of unique non-target words. |
| Task 7 supermarket selection | Cued/recognition retrieval | Touchscreen | Triggered only if free recall is incomplete. 1 point per unique target item selected via cued supermarket categories. 1-point penalty per incorrect selection. | 0-6 conditional | Count of non-target or duplicate selections. |
| Task 8 | Functional calculation | Touchscreen | Calculation of correct change (€12 from a €50 bill on a €38 purchase). 1 point per correct visual denomination combination selected. | 0-3 | Count of incorrect change options selected. |
| Task 9 | Delayed contextual recall | Numeric input | Long-term contextual retrieval of the broadcast details: 12 rescues, 400 meters, and 11 p.m. / 23:00 (1 point each). | 0-3 | Not stored. |

*Note:* The total CogniPaz score is a deterministic linear aggregation calculated as: $\text{Total Score}=\sum\left( \text{Task}_{1}\ldots\text{Task}_{9} \right)$, yielding a maximum of 38 points. Error variables are tracked independently as implementation and data diagnostics indicators and do not contribute to the total cognitive score.

# 6. Application data model summary

| Category | Examples of variables |
| --- | --- |
| Record/session | record_id, fecha, tiempo_total |
| Task scores | puntuacion1_ronda1, puntuacion1_ronda2, puntuacion2, puntuacion31, puntuacion3, puntuacion4, puntuacion5, puntuacion6, puntuacion7_ronda1, puntuacion7_ronda2, puntuacion8, puntuacion9 |
| Task errors | errores1_ronda1, errores1_ronda2, errores2, errores7_ronda1, errores7_ronda2, errores8 |
| Task times | tiempo1, tiempo2, tiempo31, tiempo3, tiempo4, tiempo5, tiempo6, tiempo7, tiempo8, tiempo9 |
| Usability | percepcion, dificultad_uso, informacion, claridad_tareas, tiempo_tareas, dificultad_tareas, utilidad, comentarios |
| Assistance | ayuda, veces_ayuda, tareas_ayuda, observaciones |
| Participant/clinical variables | edad, sexo, nivel_educativo, antecedentes, test_de_cribado, total_moca, MoCA domain scores, total_mmse, diagnostic variables and biomarker-related variables when entered |
